# Excavating the Potential of Photo‐ and Electroupcycling Platforms Toward a Sustainable Future for Waste Plastics

**DOI:** 10.1002/smsc.202300096

**Published:** 2023-11-27

**Authors:** Ling Chang, Yan Xia

**Affiliations:** ^1^ International Collaborative Laboratory of 2D Materials for Optoelectronic Science & Technology Engineering Technology Research Center for 2D Materials Information Functional Devices and Systems of Guangdong Province Institute of Microscale Optoeletronics Shenzhen University Shenzhen 518060 China; ^2^ Institute of Fire Safety Materials, School of Materials Science and Engineering NingboTech University Ningbo 315100 China

**Keywords:** electroupcycling, photoupcycling, sustainable developments, value-added chemicals, waste plastics

## Abstract

Traditionally, waste plastics have been recycled using crude recycling processes, which pose serious environmental pollution and low recycling efficiency. The best option for managing waste plastics is to upgrade them into high‐value‐added chemicals by breaking down specific chemical bonds in plastic polymers. Herein, this article reviews recent representative research advancements in photo‐ and electrodegradation of plastics for obtaining small‐molecule, high‐value‐added chemicals. The focus is on the application and analysis of emerging technological processes and catalytic materials concepts in these upcycling pathways, with particular emphasis on the diverse functions exhibited by various catalysts. Through the optimization of plastic degradation processes with the rational design of degradation technical processes and catalysts, efficient and selective access to the target products, with the assistance of light and electrical energy with similar action principles, can be achieved. These efforts have yielded promising results, paving the way for resourceful plastic recycling. Particularly, the challenges that arise in upcycling of waste plastics are seriously discussed, and reasonable recommendations have been made to understand future developments and prospects in this research area. It is believed that photocatalysis and electrocatalysis, two emerging degradation methods, play an increasingly important role in the field of plastic upcycling.

## Graphical abstract

This article reviews innovative methods to break down plastic waste using light and electrical energy, emphasizing the creation of high‐value chemicals rather than simply disposing of the waste. By optimizing degradation processes through rational design and catalysts, one can efficiently and selectively access target products using similar principles of light and electrical energy.

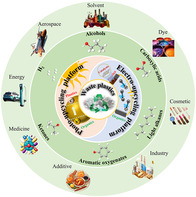

## 1 Introduction

As one of the greatest inventions of mankind, plastic has made an indelible contribution to the development of society. Large‐scale production of plastic began in the early 20th century and showed exponential growth by the mid‐20th century, with plastic production in 2014 being 20 times than in 1964.^[^ ^1^ ^]^ In 2018, global annual plastic production was reported to be around 360 million tons and the consumption of plastic products has been increasing year by year due to rapid economic development. In addition, the large‐scale use of single‐use plastic products, such as gloves and masks, as prevention equipment during the COVID‐19 epidemic in late 2019 has led to the generation of more plastic medical waste.^[^ ^2^ ^]^ It is estimated that approximately 129 billion mask and 65 billion gloves are discarded globally each month, further worsening the problem of discarded plastics.^[^ ^3^ ^]^ The rapid accumulation of plastic waste, whether buried in landfills or entering the natural environment, poses a serious economic and environmental hazard. Evidently, despite the considerable economic value of plastic packaging, estimated at ≈$100 billion per year, it is unfortunate that such value is lost globally after a single use.^[^ ^4^ ^]^ Alarming projections indicate that if present patterns persist, the weight of plastic waste in the ocean is expected to surpass that of fish by 2050.^[^ ^5^ ^]^ Plastic, which is an artificial polymer material, is difficult to recycle and tends to accumulate in the environment due to its undegradable properties.

In particular, waste plastics gradually break down microplastics (MPs), which are less than 5 mm in diameter. These MPs endure long‐term physical, chemical, and biological interactions in the environment, posing a more serious threat to the normal activities of organisms in the biosphere.^[^ ^6^, ^7^, ^8^ ^]^ The migration and transformation processes of MPs with different physical properties (e.g., material, size, structure etc.), are complex and variable, seriously affecting their circulation patterns in the biosphere.^[^ ^9^, ^10^ ^]^ The physical properties involving component and structure of MPs also have a dramatically impact on their circulation in the biosphere. Recent data have shown large amounts of plastic debris in the sea, in many regions, with most of it originating from land.^[^ ^11^, ^12^ ^]^ As most plastics are chemically stable, they are difficult to completely degrade in a short time under normal conditions. This makes the accumulation of MPs inside organisms relatively easy, which can result in the production of harmful endocrine disruptors like bisphenol A (BPA), polybrominated diphenyl ethers (PBDE), and tetrabromobisphenol A (TBBPA)^[^ ^13^, ^14^ ^]^ and can also cause birth defects and brain development risk. In addition, MPs can also pose a serious threat to the health of other plants and animals.^[^ ^15^, ^16^, ^17^ ^]^ In order to mitigate the increasing impact of waste plastics on the environment and living organisms, various methods have been developed to control and eliminate their pollution, including many traditional methods of degrading plastics, as well as new degradation platforms for MPs.^[^ ^18^ ^]^

The urgent need for strict control of both bulk plastics and MPs cannot be overstated. In particular, it is necessary, practical, and important to exert control over and degrade large plastics before they are crushed into MPs.^[^ ^19^ ^]^ Consequently, the issue of plastic degradation at the source is of fundamental importance in preventing further miniaturization of plastics. Despite this pressing need, an overwhelming number of plastics end up in landfills or natural environments. As of 2015, ≈79% of all plastics produced by humans (6300 Mt) were discarded, with only 12% being incinerated and a mere 9% being recycled.^[^ ^20^ ^]^ Both landfills and incineration have an incalculable impact on the environment. Despite the devastating ecological impact of landfilling plastic waste,^[^ ^21^ ^]^ the same fate will be arranged for mass of plastic waste, as it is the least expensive option for disposal. Incineration, is increasingly being recognized as an effective form of energy recovery. This is due to the high heat value (HHV) of plastics, which ranges between 20 and 40 MJ kg^−1^ (polyethylene glycol terephthalate (PET) and polypropylene (PP), respectively). The HHV of plastics is comparable to crude oil (45 MJ kg^−1^), making incineration an attractive option for the disposal of plastic waste.^[^ ^22^ ^]^ Furthermore, although incineration can provide a significant energy reserve, the associated environmental hazards make this method incalculable. The incineration of plastics can easily produce dioxins and other harmful compounds that are released into the atmosphere^[^ ^23^ ^]^ and generate large quantities of greenhouse gases (GHG).^[^ ^24^, ^25^ ^]^ According to statistics, nearly 1.7 gigatons of CO_2_ equivalent (CO_2_e) were immoderately emitted throughout the life cycle of plastics in 2015.^[^ ^26^ ^]^ Worryingly, with the current growth trend of plastic consumption, by 2050, it is expected that there will be about 6.5 gigatons of CO_2_e emissions, which will have a serious impact on climate change. Meanwhile, some argue that mechanical recycling of plastics should be preferred due to its lower environmental impact. While this perspective may seem reasonable at first glance, it is accompanied by a number of problems.^[^ ^27^ ^]^ The drawback of this technology lies in the varied responses of different types of plastics to the recycling process, influenced by their chemical composition, mechanical properties, and thermal characteristics. These factors hinder the broader adoption of mechanically recycled plastics.^[^ ^28^ ^]^ Aside from these mainstream ways of treating waste plastics mentioned above, other processes such as carbonization and pyrolysis,^[^ ^29^ ^]^ hydrocracking,^[^ ^30^ ^]^ and biodegradation are expected to contribute toward the elimination and recycling of waste plastics.^[^ ^31^ ^]^ These methods include using waste plastics as transparent conductive materials for solar energy production,^[^ ^32^ ^]^ carbon materials for seawater desalination,^[^ ^33^ ^]^ electrode materials for lithium‐ion batteries,^[^ ^34^, ^35^ ^]^ and carbon nanotubes (CNTs),^[^ ^36^ ^]^ among others. However, recycling processes that involve high‐energy consumption, harsh transformation conditions, or deteriorating properties of the recovered material are usually considered undesirable due to the obstacles they present to plastics recycling. Therefore, it is promising to design and develop new technological processes to construct a more economical and convenient platform for the upcycling and recycling of waste plastics.^[^ ^37^, ^38^ ^]^ A number of new plastic degradation technologies are flourishing and expected to become effective means of recycling waste plastics.^[^ ^39^, ^40^, ^41^ ^]^ For example, waste plastics could be transformed into high‐value‐added chemicals through the intervention of light or electricity, thus achieving the resource recycling of used plastics.

In recent years, many researchers have expressed dissatisfaction with the traditional methods of plastic waste degradation and instead are exploring ways to transform waste plastic into chemicals of higher value. This process is known as “upcycling or resource‐based recycling” and aims to turn waste plastics into a valuable resource through resource recycling.^[^ ^42^, ^43^, ^44^ ^]^ Upcycling can provide economic, environmental, and social benefits by turning waste plastics into higher‐value products. One promising approach for plastics upcycling is the photocatalytic degradation technology to obtain higher chemical value products.^[^ ^45^, ^46^, ^47^ ^]^ Photocatalysts can break down polymer compounds under mild conditions, even at room temperature and ambient pressure,^[^ ^48^ ^]^ by generating holes/electrons generated from light, and electrical input can drive a serious of chemical reactions.^[^ ^49^ ^]^ By properly regulating the tank pressure of the electrolytic cell and adjusting the energy band structure of the photocatalyst, it is extremely possible to cleave the specified chemical bonds and achieve high selectivity of chemical products. For example, in the presence of a catalyst, polymers can undergo degradation through light irradiation, yielding aromatic small‐molecule chemical products,^[^ ^50^, ^51^ ^]^ short‐chain oligomers, polymer monomers,^[^ ^52^ ^]^ and even simultaneously producing H_2_ fuel,^[^ ^53^ ^]^ which can be further utilized for the crude production of chemical products. This way, both plastic waste degradation and clean energy preparation can be achieved. Though research into electrocatalytic degradation of plastics is still in its initial stage, it has shown the rapid development potential in the future. Resourceful recycling of photo‐ and electrocatalytic platform for plastics degradation has become a new frontier research area and has garnered broad attention from the scientific community.

In previous studies, the products of photo‐ and electrocatalytic plastic degradation were mainly CO_2_.^[^ ^54^ ^]^ However, converting plastics waste into fuels and low‐carbon organic compounds through photo‐ and electrodegradation is more in line with the concept of circular economy. In recent years, nonthermal processes of photo‐ and electrodegradation of waste plastic and resource‐based upcycling have received great attention.^[^ ^55^ ^]^ Currently, there are already numerous outstanding reviews available that provide in‐depth discussions on plastic recycling and plastic waste management. Many of them primarily emphasize the processes of plastic pyrolysis and solvolysis.^[^ ^42^, ^56^, ^57^, ^58^ ^]^ However, in recent years, there has been significant growth in academic advancements, particularly in catalytic chemical transformations.^[^ ^48^ ^]^ Consequently, in this review, our focus lies on the effect of different types of catalysts, along with the introduction of two upcycling techniques: photo‐ and electroupcycling processes, especially the latter, for which there is scarce literature review. Furthermore, recommendations are provided for enhancing catalysts and the associated technological processes. Here, we take recent representative cases with innovative materials and technological processes as the pointcut to discuss photo‐ and electrodegradation for plastics upcycling, respectively, and involving the coupling of thermal assistant. Although photo‐ and electroupcycling technologies are still in their infancy in resource‐based upcycling of waste plastics, we believe that new technological processes and catalyst designs will be key in addressing existing scientific challenges. Hence, we categorize and review these recent catalytic pathways that aim to tackle plastic upcycling dilemmas in terms of emerging technological process development and catalysts design, respectively.

**Figure 1:**
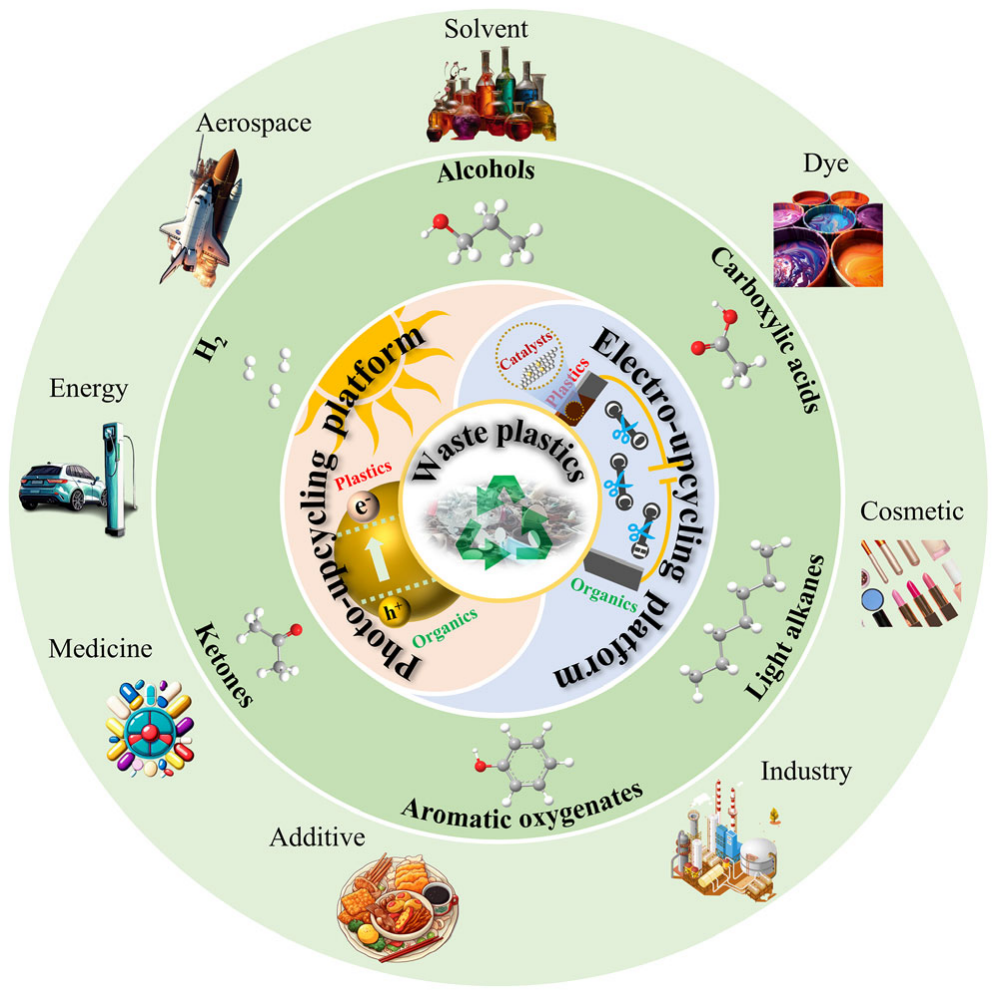
Overview of waste plastics conversion into high‐value‐added products by photo‐ and electroupcycling platforms.

We will be focusing on the latest advancements in chemical recovery and upgrade pathways in the field of photo‐ and electroupcycling for the production of high‐value‐added chemicals and fuels from waste plastics (**Figure** **1**). This will include direct degradation of waste plastics into monomers, monomer derivatives, oligomers, and hydrogen fuels. Additionally, we'll explore ways to access CO_2_ for further upcycling into C1 or C2 small molecules such as methane, formic acid, methanol, ethane. Using upcycling of waste plastics within the context of artificial photosynthesis, we hope to tackle the problem of CO_2_ GHG emissions. Furthermore, the potential of clean energy H_2_ fuel adds further appeal to the process of upcycling waste plastics.

From this perspective, postconsumer waste plastics are not insufferable pollutants, but in fact raw materials composed of hydrocarbon‐rich compounds. Upcycling these waste plastics can be viewed as a valid means of converting solar and secondary electrical energy into chemical energy for stable storage and convenient usage. Increasing the available value of postconsumer plastic waste products provides an economically and environmentally sustainable solution to the postdisposal problem, while simultaneously reducing the heavy dependence on fossil energy sources, such as oil. The purpose of this review is to shed light on the development of catalytic materials and innovative processes to overcome the economic barriers that arise in the upcycling process of waste plastics.

## 2 Photoupcycling Platform

Solar energy is the most common source of infinite clean energy that brings endless well‐being to the reproduction of life. However, as a source of energy, it can also contribute to the aging and degradation of plastics. As a result, people often take precautions to protect plastic materials from light damage to ensure that they remain useful for as long as possible. When plastic materials have outlived their usefulness, they can be transformed into useful small‐molecule compounds through the process of waste plastic or biomass degradation with the aid of light energy.^[^ ^59^ ^]^ Indeed, in addition to degradation polymers into high‐value‐added small molecules, there is another way to convert waste plastics into useful products by modifying postconsumer plastics through changes in its surface tension.^[^ ^60^ ^]^ For example, fluoroalkylation is used to modify postconsumer PS plastics by generating electrophilic fluoroalkyl radicals in a photocatalytic process, creating a more hydrophobic surface.^[^ ^61^ ^]^ However, such upgraded products are difficult to recycle, so we do not dwell on such work here. Photoenergy‐driven plastic degradation reactions could convert waste plastics into high‐value‐added products under mild conditions which have considerable economic benefits.^[^ ^62^ ^]^ Light promotion of waste plastics degradation includes photochemical oxidative degradation, photocatalytic degradation, and photothermal degradation, which have gained significant attention from researchers worldwide in recent times.

### 2.1 Photochemical Oxidative Degradation

Photochemical oxidation generally refers to the process of oxidation reaction that occurs when a light source stimulates the oxidant or acts directly on the plastic without photosensitizers involved. This can lead to the splitting of the chemical bonds of the polymer, including light‐induced polymer chain breakage with the participation of impurities in or out. Therefore, light stimulation‐induced chemical bond cleavage is considered a key process in polymer degradation.^[^ ^63^ ^]^ Long‐term exposure to ultraviolet (UV) light with high energy could lead to the breakage or crosslinking of polymer chains by free radical erosion, oxygen incorporation, and hydrogen extraction,^[^ ^64^ ^]^ which is known as photo‐oxidation, chain scission, crosslinking, and secondary reactions.^[^ ^65^ ^]^ In addition, peroxides, oxygenated chromophores, carbonyl groups, and catalyst residues inside plastics could be activated by light to produce reactive groups, thereby hastening the chemical oxidation of polymers.^[^ ^66^ ^]^ This section introduces various photo‐oxidation systems that facilitate the cleavage of C—C, C—O, and C—N bonds.

#### 2.1.1 Homochain Polymers

**Figure 2:**
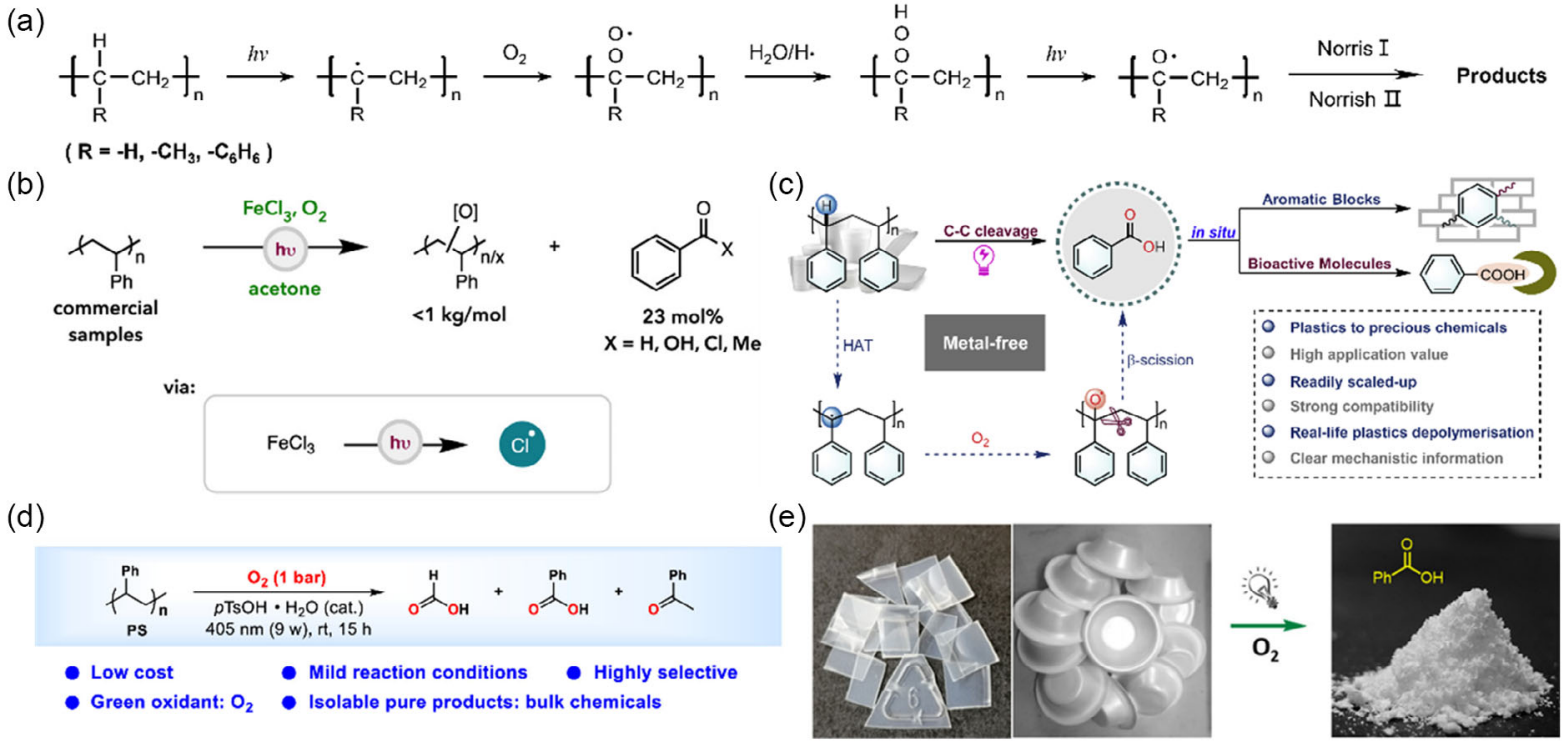
Photo‐oxidative degradation pathway of a) PE, PP, and PS.^[^ ^68^ ^]^ Copyright 2005, Wiley‐VCH. b) Degradation of PS by chlorine radicals for HAT involving ferric chloride.^[^ ^80^ ^]^ Copyright 2022, American Chemical Society. c) Metal‐free HAT degradation of PS and subsequent high‐value compound synthesis.^[^ ^82^ ^]^ Copyright 2022, Cell Press. d,e) Photodegradation of PS domestic waste under acidic conditions.^[^ ^81^ ^]^ Copyright 2022, American Chemical Society.

Polyolefin plastics, such as PP, PE, PS, and polyvinyl chloride (PVC), which have a chemically stable C—C bond as the main chain, are widely consumed in our daily lives. Theoretically, when the radiation energy of UV light is strong enough, which is more than three orders of magnitude higher than the bond energy of C—C (375 kJ mol^−1^) and C—H (420 kJ mol^−1^) bonds, it can directly cause the cleavage of these chemical bonds and then attack the adjacent chemical bonds through free radicals.^[^ ^67^ ^]^ However, it is difficult for natural light to directly initiate the degradation of highly stable C—C bond‐dominated plastics like PE and PP. Simulating the degradation of pure PE and PS under sunlight did not yield a detection of the free radical signal on the surface of the plastic.^[^ ^64^ ^]^ Thus, it can be concluded that the natural degradation of PE, PP, and PS plastics in nature by sunlight is mostly induced by their internal impurities during the degradation process (**Figure** **2**a).^[^ ^68^ ^]^ These impurities are stimulated by light to generate free radicals that directly attack the C—H bond in the plastic, forming alkyl radicals (R^•^) and free hydrogen (H). The highly reactive R^•^ species reacts with the surrounding oxygen to form more oxidized peroxy radicals (ROO^•^). These radicals then combine with hydrogen in the plastic to form hydroperoxide groups (ROOH), which improve the stability of the chemical groups. The ROOH is further cleaved to alkoxy (RO^•^) and hydroxyl radicals (HO^•^).^[^ ^64^ ^]^ At the same time, the HO^•^ attacks the C—C and C—H bonds of the polymer. This leads to the degradation of the polymer into oligomers or small molecules following the chemical bond breakage. The production of HO^•^ is usually considered as the main cause of the oxidative degradation of the polymer.^[^ ^69^ ^]^ The RO^•^ will eventually be converted into carboxylic acids, aldehydes, ketones, and other oxygen‐containing unsaturated functional groups through Norrish Type I and Norrish Type II. These groups continue to absorb photons and generate free radicals, causing the running of polymer chain breakage reaction.^[^ ^70^ ^]^ Generally, for plastic polymers with C—C bond as the main chain, the secondary carbon on the PE polymer chain is more stable than the tertiary carbon attached to the methyl group on the PP molecular chain. Similarly, the tertiary carbon of PS attached to the phenyl functional group is more vulnerable to the attack of free radicals than that on PP, and PVC containing heteroatoms is more easily degraded than PS. Therefore, in general, C—C chain‐based polymer plastics are ranked according to stability as PE > PP > PS > PVC.

Plastic degradation, in any form, is essentially the cleavage of polymer chains. The amount of energy required for chemical bond breaking heavily depends on the chemical bond types present in different genres of plastics. The cleavage of chemical bonds by external energy injection in the presence of oxidizing agent is a prevalent research field for polymer degradation. For instance, the degradation of PS is challenging to catalyze under normal Fenton reaction conditions and requires the aid of additional light.^[^ ^71^ ^]^ Plastics like PE, PP, and PS are often challenging to degrade under mild conditions and typically require the involvement of oxidants and more stringent reaction conditions for efficient degradation.

The photo‐Fenton reaction is a commonly useful method for solving plastic problems. The chemical reactions involved in this method are strong oxidation reactions, known as advanced oxidation processes (AOPs), which are effective for cleavage stable C—C bonds, leading to the rapid degradation and mineralization of highly stable chemicals.^[^ ^72^, ^73^ ^]^ This oxidation approach is carried out by two main processes, similar to Equation (1), (2) and (3), which enable the homolysis fission of H_2_O_2_ to produce hydroxyl radicals (HO^•^, H_2_O/HO^•^ = 2.22 V vs normal hydrogen electrode (NHE)),^[^ ^74^ ^]^ and reactive oxygen species (ROS) with strong oxidizing properties that break C—C bonds, eventually leading to mineralization in the presence of variable metals such as Fe, Co, Cu, etc. For example, the Fenton reaction, in the dark, can convert PP to water‐soluble organic matter and CO_2_ in 40 min, while the photo‐Fenton reaction under UV–vis illumination achieves complete mineralization of PP in 80 min.^[^ ^75^ ^]^ Although a wide range of highly stable polymers could be degraded by means of photo‐Fenton until mineralization into small CO_2_ molecules, this degradation pathway, which involves high‐energy consumption without available material recovery, is not well accepted. For instance, sulfonic acid groups (–SO_3_^−^) were modified on the surfaces of PE, PP, and PVC by chlorosulfuric acid, and then Fe (III) catalysts were grafted onto the polymers to obtain active polymers. These functional polymers were then converted into thermal (1800–3200 J g^−1^) and mechanical energy (380–560 kPa g^−1^) using H_2_O_2_ as the oxidizing agent under light irradiation condition.^[^ ^76^ ^]^ This way of degrading postconsumer plastics to obtain profit gain via a certain energy payment is considered a very promising pathway for plastics upcycling. Although there is no guarantee that the profit gain is greater than the consumption, this approach to waste plastics disposal will certainly give us food for thought.

(1) $${\left(\text{Fe}-\text{OH}\right)}^{2+}+\text{hv}\to {\text{Fe}}^{2+}+{\text{HO}}^{•}$$

(2) $$\text{Fe}\left(\text{III}\right)\left({\text{RHCO}}_{2}\right)+\text{hv}\to {\text{Fe}}^{2+}+{\text{CO}}_{2}+{\text{RH}}^{•}$$

(3)

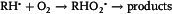




Compared to PP, PE, and other polymer plastics with high chemical stability, PS is much easier to degrade. Even under freezing conditions, PS can experience carbon chain breakage due to solar exposure.^[^ ^77^ ^]^ Prolonged exposure to sunlight can also cause aging of PS in water. This is caused by ROS radicals such as monomorphic oxygen (^1^O_2_, ^1^O_2_/O_2_ = 1.88 V vs NHE),^[^ ^78^ ^]^ hydroxyl radicals (HO^•^), and superoxide radicals (^•^O_2_^−^, O_2_/^•^O_2_^−^ = −0.33 V vs NHE).^[^ ^59^ ^]^ ROS radicals originate from a series of chemical reactions between the initiating radicals on the PS surface and water molecules or oxygen and are responsible for breaking the C—C and C—H bonds on the polymer surface.^[^ ^79^ ^]^ While it is possible to degrade PS to some extent without much interference, this does not achieve our desired goal. Moreover, in nonaqueous environments, such as acetone, FeCl_3_ generates chlorine radicals under white light to extract electron‐rich hydrogen atoms from the PS backbone, thus reducing the stability of PS chains (Figure 2b). In an oxygen‐rich environment, this process can degrade high‐molecular‐weight PS (>90 kg mol^−1^) to oligomers of less than 1 kg mol^−1^ while producing up to 23 mol% benzoyl products.^[^ ^80^ ^]^ While the pathway of controlled degradation of polymer into small‐molecule products by light driving will be our research direction in the future, these results show that this method cannot degrade PS completely and the products are more complex and diverse. The oxidative degradation of PS can also be achieved by visible light irradiation mediated by acidic substances. It has been shown that O_2_ plays an important role in this degradation process as ROS, which extracts a hydrogen atom from the tertiary C—H bond, leading to hydrogen peroxidation and C—C bond cleavage. This ultimately yields small‐molecule products such as formic acid, benzoic acid, and acetophenone, among others (Figure 2d,e).^[^ ^81^ ^]^ Photoinduced ligand–metal charge transfer (LMCT) produces chlorine radicals, which are utilized for the hydrogen atom transfer (HAT) process to generate benzyl radicals. O_2_ is involved in the formation of O radicals on the PS backbone, which subsequently undergo β‐split/α‐bond splitting to form intermediates. The highly reactive intermediate is further oxidized by the oxygen radical to give the final product benzoic acid (Figure 2c).^[^ ^82^ ^]^ This idea of triggering the oxidation and degradation of plastic polymer to small molecules by photostimulation suggests a research direction for the resourceful upcycling of waste plastics.

Therefore, the light‐driven C–C cracking reaction using homogeneous catalysts and O_2_ as an oxidant could be utilized for resource recovery of plastic waste^[^ ^83^ ^]^ or in turn, it could be transformed into fuel and chemical feedstock.^[^ ^84^ ^]^ These homogeneous oxidation systems are particularly beneficial in plastic upcycling and recycling applications, specifically in cases where the C—C bond is the main chain.

#### 2.1.2 Heteroatomic Main Chain

Compared to polyolefin plastics with C—C bonds as the main chain, polymers with heteroatoms in the main chain, including polylactic acid (PLA), an aliphatic polyester plastic, and polyurethane PU, an amide ester polymer, are significantly easier to degrade. Among these, PLA, with C—O bonding as the main chain, has seen a sharp increase in production for industrial applications, including medical, textile, packaging products, and environmental remediation, due to its competitive cost and positive public awareness of environmental protection.^[^ ^85^ ^]^ Even though PLA is considered subordinate to biodegradable materials, it has a long degradation cycle, making it difficult to reflect all of its biodegradable advantages over a short period of time under normal conditions. Nonetheless, it does not lead to excessive accumulation of postconsumer plastics.^[^ ^86^ ^]^

**Figure 3:**
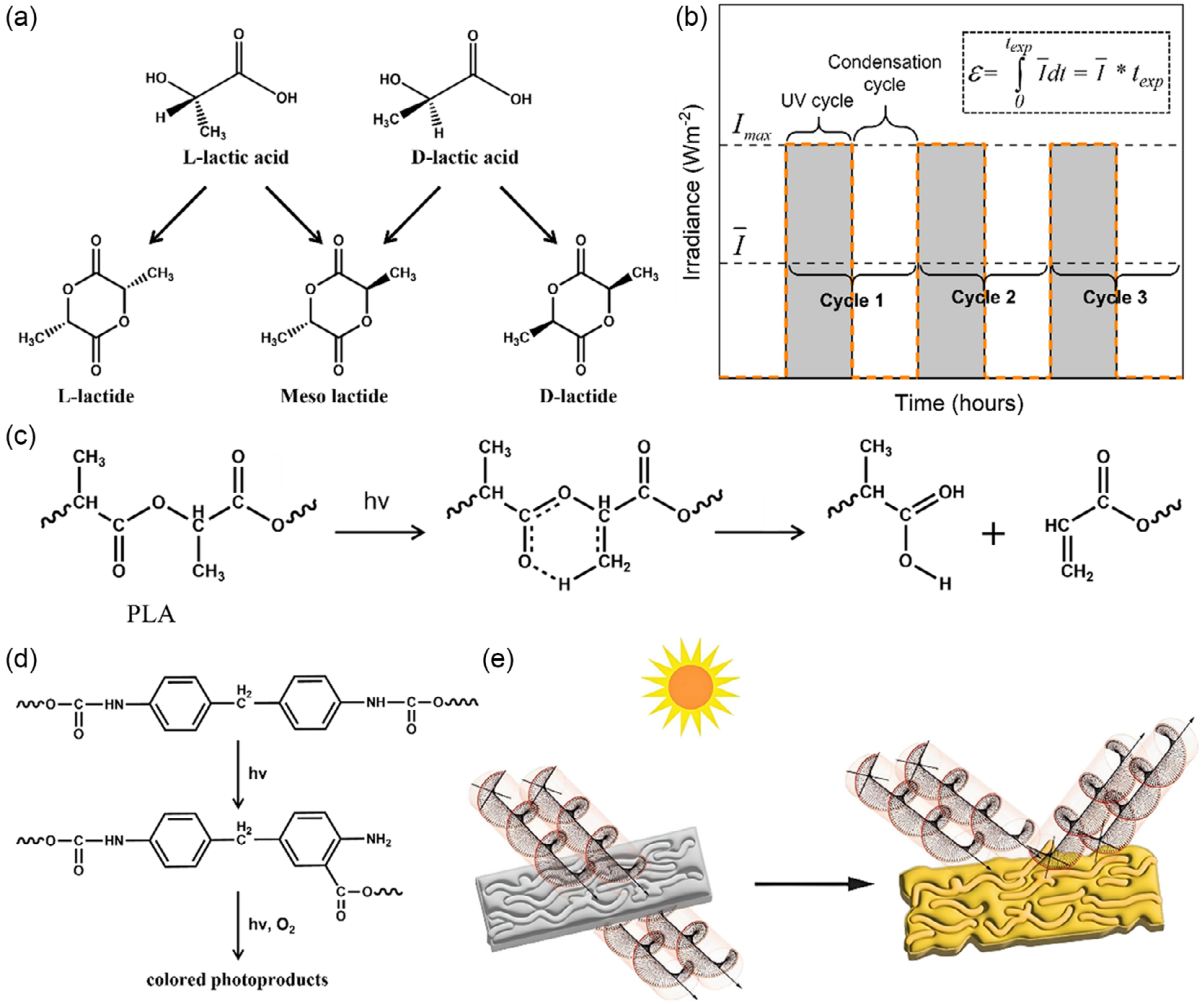
a) Stereoscopic configuration of lactides.^[^ ^87^ ^]^ Copyright 2010, Elsevier Ltd. b) Calculation of total absorbed energy during plastic degradation.^[^ ^89^ ^]^ Copyright 2020, Elsevier Ltd. c) Photodegradation of PLA via the Norrish II mechanism.^[^ ^88^ ^]^ Copyright 1993, Tokai University. d) Photo‐Fries‐type mechanism in MDI‐based polyurethanes.^[^ ^94^ ^]^ Copyright 2007, Royal Society of Chemistry. e) The chiral supramolecular crystal structures are formed on the surface of the aged PE film due to chemical conversion.^[^ ^97^ ^]^ Copyright 2005, Wiley‐VCH.

Under light, the PLA backbone undergoes photodegradation via the Norrish II mechanism, where electron transfer of the C=O bond on the backbone leads to the formation of a C=C bond at the carboxyl end (**Figure** **3**a,c),^[^ ^87^, ^88^ ^]^ while the degradation rate depends on the total absorbed energy during UV irradiance (Figure 3b).^[^ ^89^ ^]^ The degree of decomposition of plastics during photo‐oxidative degradation of plastics depends on the energy input.^[^ ^89^ ^]^ While the breakage of the backbone during photo‐oxidative degradation is random, the probability of photodegradation is higher in the amorphous region compared to the crystalline region. The presence of anhydride groups in the polymer structure leads to a decrease in the molecular weight (Mw) of the polymer.^[^ ^90^ ^]^ The presence of carbonyl groups in the PLA molecular structure means that it can absorb UV radiation at around 280 nm by n–π* electron leap, which makes PLA more sensitive to UV light.^[^ ^91^ ^]^ Therefore, PLA is highly susceptible to degradation when exposed to low‐wavelength and high‐energy UV radiation from sunlight during its entire lifespan, whether as plastic cultivation, packaging containers, or films. Other mechanisms predicting PLA degradation under UV irradiation have been proposed.^[^ ^92^ ^]^ In addition, gamma (γ) radiation (γ‐rays), with its high penetrating ability, can cleave PLA ester bonds, and γ‐radiation degradation occurs mainly in the amorphous region of the polymer. Electron spin resonance (ESR) spectroscopy was used to investigate the effect of γ radiation, where the free radicals formed during degradation result from the breakage of ester bonds in the PLA backbone and the hydrogen extraction of methane groups.^[^ ^93^ ^]^

Furthermore, amide ester polymers containing more heteroatoms (N, O) in the main chain are sometimes more prone to breakage compared to the C—O bond. The presence of heteroatoms in molecular chains, when subjected to light irradiation, can potentially result in frequent electron transfer and heightened instability (Figure 3d).^[^ ^94^ ^]^ Aromatic PU polymers containing benzene rings generally have more complex photodegradation mechanisms relative to aliphatic polymers. Although the resulting products are more difficult to separate, they are more susceptible to photo‐oxidative degradation.^[^ ^68^ ^]^ When irradiated at light wavelengths less than 340 nm, phenyl undergoes photo‐Fries rearrangement, and at wavelengths greater than 340 nm, phenyl can form quinone imides. The formed quinone‐like structure is thought to be responsible for the typical yellow color observed during the photodegradation of aromatic PU,^[^ ^95^ ^]^ and the photo‐Fries reaction may also contribute to the color change.^[^ ^96^ ^]^ In addition, the chiral supramolecular structure on the polyethylene (PE) surface can lead to the light change (Figure 3e).^[^ ^97^ ^]^ The difference in color shades of plastics due to photo‐oxidative degradation is most likely related to the concentration of carbonyl groups in the plastic.^[^ ^95^ ^]^ Further light irradiation on this basis leads to random breakage of polymer chains through the Norrish type I mechanism, resulting in a reduction of molecular weight. For example, in the study of the photodegradation mechanism of PU synthesized from diphenylmethane‐4,4′‐diisocyanate (MDI) and polyester from adipic acid and ethylene glycol (EG), initial irradiation formed quinone structures. Upon further irradiation with light, these structures triggered chemical bond breakage and free radical generation in the polyurethane moiety.^[^ ^98^ ^]^ The free radicals attack the intramolecular atoms to form nitrogen (II), amino (III), and carbodiimide (IV) structures. At the same time, oxygen molecules react with the free radicals to obtain hydroperoxides, the decomposition of which leads to the formation of carbonyl‐containing structures (I). The final polymer PU degrades into molecules with various chemical functional groups such as carbonyl, carbodiimide, azo, and amino.

Similarly, plastics with heteroatoms in the main chain could be oxidatively degraded under light irradiation conditions. However, recovering monomers or derivative products for resource‐based upcycling may be challenging. Although the photo‐oxidative degradation of olefin polymer plastics can reduce them to low‐molecular‐weight chain segments by light irradiation, this process simply results in “disintegration” without actual degradation into high‐value‐added small‐molecule chemicals or environmentally benign substances.^[^ ^99^, ^100^ ^]^ Furthermore, small fragments resulting from disintegration remain in the environment and cannot be recycled, which affects the growth of plant roots and may even enter the human body through the food chain, posing health risks.^[^ ^101^, ^102^ ^]^ The generation of free radicals during photo‐oxidative degradation is also more difficult without a catalytic medium to trigger the reaction process. Photo‐oxidative degradation is an extremely slow process, especially in an aqueous environment. Under low radiation and low‐temperature conditions, the natural degradation rate of polymers is even lower and less effective. Thus, without added catalysts, the upcycling of waste plastics through light irradiation is unlikely a viable resource‐based solution.

### 2.2 Photocatalytic Degradation

Photocatalytic technology has been widely used in various scientific research fields.^[^ ^103^, ^104^, ^105^, ^106^ ^]^ However, the upcycling of waste plastics through photocatalysis is still in its initial stage. Utilizing solar energy to convert waste plastics into high‐value‐added chemicals or fuels would be a sustainable, economical, and environmentally friendly approach. The resourceful photocatalytic degradation of plastics can eliminate the large amount of waste plastics that accumulate while generating useful chemical products by triggering redox reactions. It is unquestionable that the catalyst plays the most significant role in the photocatalytic reaction.^[^ ^107^ ^]^

Photocatalytic degradation generally refers to the separation of hole (h^+^)/electron (e^−^) pairs in the semiconductor photosensitizer, which is excited by photon energy (E). When e^−^ in the valence band (VB) of the semiconductor catalyst is transferred to the conduction band (CB), it produces h^+^ in the VB.^[^ ^108^ ^]^ These two substances (e^−^ and h^+^) react with OH^−^, O_2_ or H_2_O to produce large amounts of ROS, including superoxide radicals (^•^O_2_^−^, O_2_/^•^O_2_^−^ = −0.33 V vs NHE),^[^ ^59^ ^]^ singlet oxygen (^1^O_2_, ^1^O_2_/O_2_ = 1.88 V vs NHE),^[^ ^78^ ^]^ hydroxyl radicals (HO^•^, H_2_O/HO^•^ = 2.22 V vs NHE), etc.^[^ ^109^ ^]^ The photocatalytic reaction usually consists of three steps: 1) photo absorption of photocatalysts for exciting carrier generation; 2) separation and transport of photogenerated carriers; 3) redox reaction on the catalyst surface. The ROS attack on plastic polymers directly triggers the breakage of polymer molecular chains and even complete mineralization, resulting in the production of CO_2_.^[^ ^110^ ^]^ However, committing to the goal of upcycling of postconsumer plastics to ultimately generate CO_2_ GHG is clearly inappropriate. Many researchers have been improving catalysts in various ways to optimize the catalytic pathway and synthesizing new materials that can achieve high catalytic efficiency while avoiding excessive oxidation to produce GHG. Therefore, the latest research progresses are reported in this subsection by focusing on catalysts in various categories, single‐component, multicomponent catalysts, heterojunction catalysts, and single‐atom catalysts, respectively.

#### 2.2.1 Single‐Component Catalysts

UV‐response wide‐bandgap semiconductors, such as TiO_2_,^[^ ^111^ ^]^ ZnO,^[^ ^112^ ^]^ and Nb_2_O_5_,^[^ ^59^ ^]^ have been widely used in previous studies for photocatalytic degradation of plastics. However, these catalysts have low solar energy collection efficiency, and the photocatalytic degradation process generates a large amount of CO_2_, which is controversial due to the greenhouse effect.^[^ ^113^ ^]^ Although CO_2_ could be further upgraded to high‐value‐added chemicals by reduction and C–C coupling, this pathway is more energy intensive and difficult to achieve economic balance.^[^ ^114^ ^]^ While the use of catalysts can significantly increase the reaction rate during plastic degradation, there is also a pressing need to develop effective catalysts for the conversion of plastics into high‐value‐added products. Polyolefin plastics, which account for more than half of global waste plastics, are a particular focus for degradation and upcycling efforts. Unfortunately, due to their chemical stability and poor solubility, it remains challenging to make them available for resource recycling by cleaving the C—C bonds on the main chains.^[^ ^115^ ^]^ Consequently, in the field of photocatalytic upgrading and recycling of plastics, polyolefin plastics have been the primary focus of attention.

To reduce the catalytic capacity of certain oxide catalysts, coating their surface with inert materials is a viable strategy to consider. There is evidence that C cladding can significantly reduce the catalytic degradation capacity of TiO_2_, which is a high‐capacity catalyst.^[^ ^116^ ^]^ In addition, the catalytic capacity of TiO_2_ was severely reduced by the tight coating of graphene, which only causes a small degree of damage to PP under solar light irradiation.^[^ ^117^ ^]^ This approach would be a way to reduce the CO_2_ yield during the process of recovering resources from plastics.

**Figure 4:**
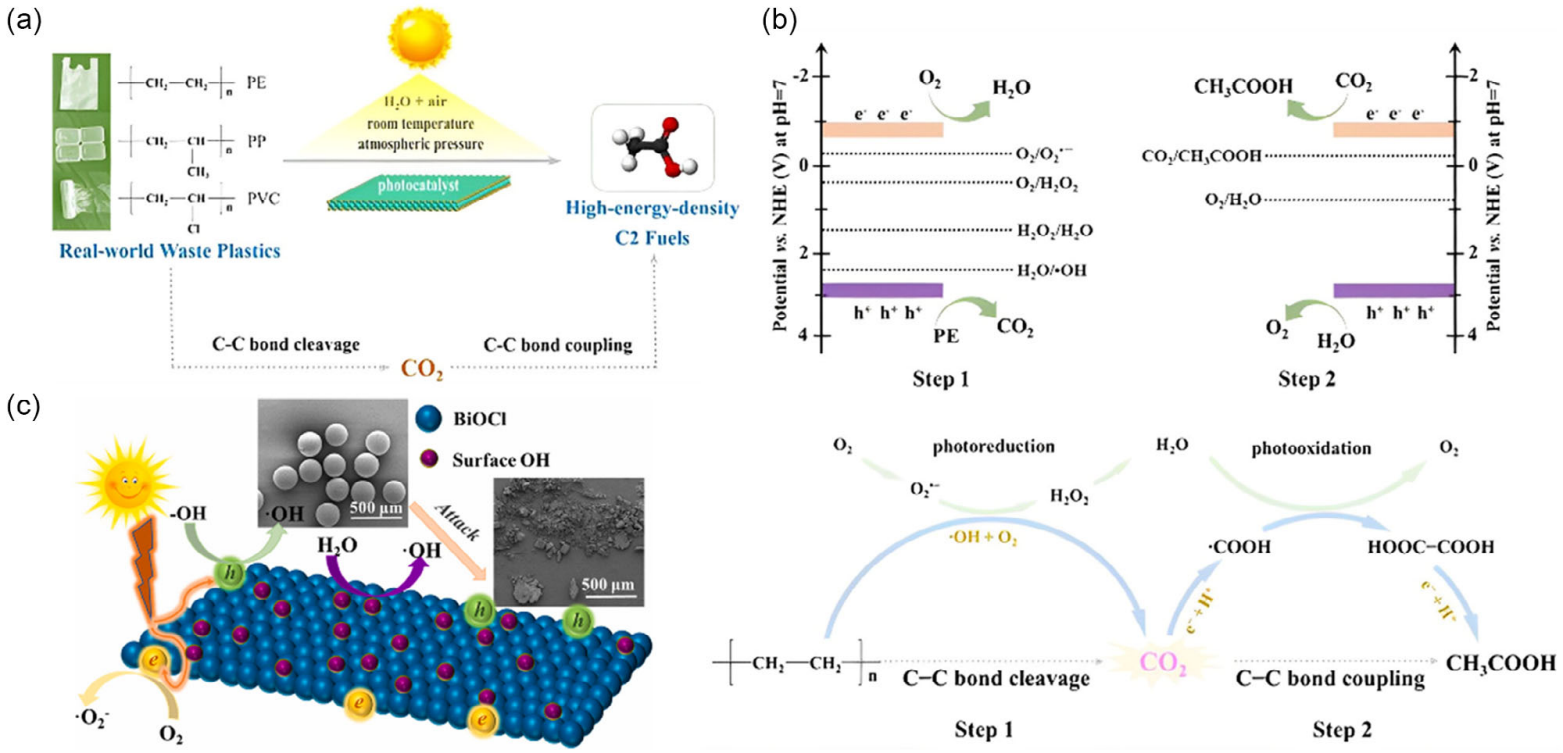
Schematic illustration of a) converting various waste plastics into C2 fuels via a designed two‐step pathway under simulated natural environments. b) The band‐edge positions of Nb_2_O_5_ catalyst along with the potentials of different redox couples (up), and the proposed two‐step C—C bond cleavage and coupling mechanism under simulated natural environments (down).^[^ ^59^ ^]^ Copyright 2020, Wiley‐VCH. c) Schematic diagram for MP degradation by hydroxy‐rich bismuth oxychloride.^[^ ^114^ ^]^ Copyright 2021, Elsevier Ltd.

Although the aforementioned photocatalysts do not fully meet the criteria for resourceful upcycling of waste plastics, some of them are still capable of obtaining small‐molecule products with economic value by degrading plastics due to the diversity of products. A study reported the design of a two‐step conversion strategy for C—C bond cleavage and coupling with Nb_2_O_5_ photocatalyst, first mineralizing PE, PP, and PVC to CO_2_ and further coupling reduced CO_2_ to form CH_3_COOH (<40.0%) by light (**Figure** **4**a,b).^[^ ^59^ ^]^ However, the poor CO2 reduction capacity of existing photocatalysts led to a low yield of the reduced products. Therefore, the optimization of C—C bond cleavage and coupling by rational design of photocatalysts is also a way to achieve the conversion of waste plastics into high‐value chemicals. In order to obtain catalysts with better photodegradability, a novel hydroxyl‐rich ultrathin BiOCl nano‐catalyst was prepared, and the mass loss of PE plastics was verified to be 24 times higher than that of ordinary BiOCl nanosheets (Figure 4c).^[^ ^114^ ^]^ Although this catalyst could enable plastics to degrade efficiently, unfortunately, the products of the degradation process, which rely heavily on hydroxyl radicals, are still dominated by CO_2_. It is clear that oxide semiconductor photocatalysts with a larger bandgap can degrade plastics well, but the products are generally low‐grade materials and hardly become important members in future upcycling processes of waste plastics.

**Figure 5:**
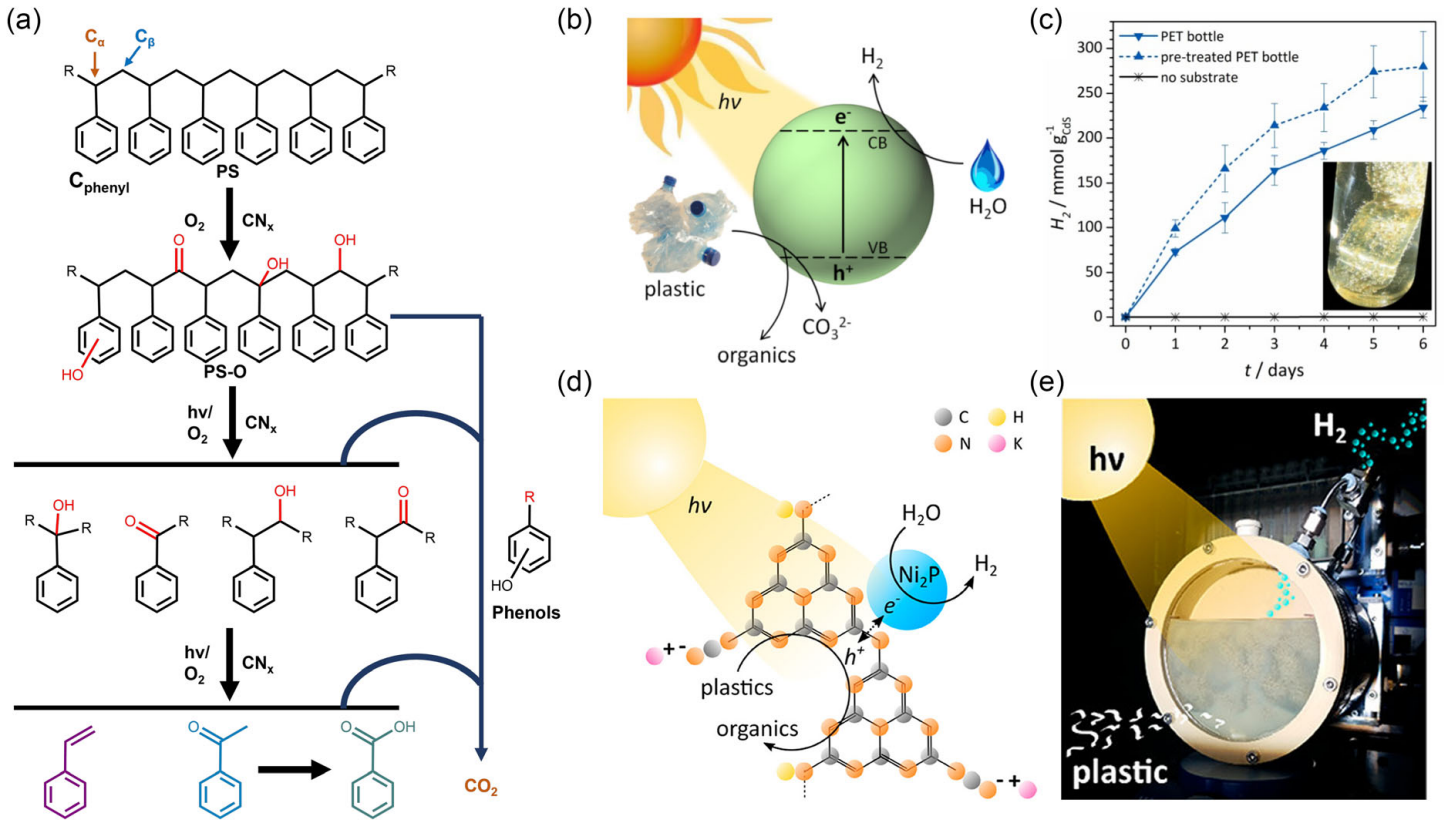
a) Proposed reaction procedure of PS photodegradation reaction processes.^[^ ^50^ ^]^ Copyright 2022, Nature Portfolio. b) Scheme of the plastics photocatalysis process with the CdS quantum dots catalyst. c) Long‐term photocatalysis of a PET bottle segment to H_2_ fuel utilizing CdS quantum dots (QD) photocatalyst under simulated sunlight.^[^ ^121^ ^]^ Copyright 2018, Royal Society of Chemistry. d) Schematic diagram of the plastics photocatalysis process using the CN_x_/Ni_2_P photocatalyst for H_2_ and organics produced. e) Photograph of the batch reactor in use.^[^ ^53^ ^]^ Copyright 2019, American Chemical Society.

The fact that UV light only accounts for a small percentage of sunlight is a major reason why solar energy is currently underutilized for plastics conversion.^[^ ^118^ ^]^ To achieve the goal of upcycling plastic into high‐value small‐molecule compounds, it is necessary to develop more efficient photocatalytic semiconductor materials that can be activated by visible light. These materials should have a smaller bandgap and weaker oxidation capabilities. Carbon nitride (CN_*x*_) with the advantage of being cheap and nontoxic is a visible light‐driven photocatalyst that has become a star material in the field of photocatalysis in recent years.^[^ ^119^ ^]^ Graphitic carbon nitride catalysts, for instance, have been used to oxidize PS degradation into aromatic compounds under visible light irradiation. The oxidation of C—H bond leads to the formation of hydroxyl and carbonyl groups on the main chain of polystyrene, and then the C—C bond is broken by oxidative activation to obtain further oxidation of small‐molecule compounds with benzoic acid, acetophenone, and benzaldehyde as the main products (**Figure** **5**a).^[^ ^50^ ^]^ Although the degradation of PS by graphitic carbon nitride catalysts under visible light has been achieved and a series of small‐molecule compounds were obtained, there was still a significant fraction of CO_2_ in the degradation products and some plastics were degraded to oligomers only rather than small molecules. Nevertheless, the use of CN_*x*_ photocatalysts for plastic degradation is still advantageous because of its low cost and its ability to recover some small molecules under visible light.

As shown, semiconductor catalysts with wide bandgap and high oxidation capacity have the ability to easily mineralize polymers into smaller molecular weight chemicals, and in most cases, catalysts with hydroxyl radical generation capability will cause plastics to produce CO_2_. Therefore, photocatalytic semiconductors with a relatively small bandgap are of interest to us. Metal chalcogenides materials generally have a small bandgap, for example, CdS (*E*_g _= 2.4 eV) possesses the ability of visible light absorption and catalytic reaction triggered without the assistance of noble metals.^[^ ^120^ ^]^ H_2_ production for PET, PLA, and PU plastics was achieved with CdS quantum dots in alkaline aqueous solutions (10 m KOH or NaOH), resulting in small‐molecule compounds (Figure 5b,c).^[^ ^121^ ^]^ Although this is an efficient catalyst in the visible light range, Cd‐based catalysts tend to be toxic, making their commercial application on a large scale challenging. Therefore, it would be fascinating to design and produce nontoxic catalysts with excellent photo‐response in the visible and potentially even near‐infrared light range. In addition, a variety of noble metal catalysts with plasmon resonance synergistic photocatalysis are potential catalysts for hydrogen production or small‐molecule compounds.^[^ ^122^ ^]^ The electron plasma generated by plasmon resonance interacts with O_2_ to generate ^•^O_2_^−^, which further abstracts hydrogen atoms from polymerization chains to form hydrogen peroxide groups, thus leading to polymer chain cleavage.^[^ ^123^ ^]^

#### 2.2.2 Multicomponent Catalysts

In comparison to single‐component catalysts, multicomponent catalysts possess the potential to fully exploit synergistic effects and enhance catalytic activity and stability. In addition to this, the composition and structure of such catalysts are more diverse, giving rise to possibilities of specific designs aimed at improving selectivity in catalytic reactions. In recent years, it has been reported that a cheap and nontoxic photocatalyst, cyanamide‐functionalized CN_*x*_ loaded with Ni_2_P, has been used to effectively promote charge separation, resulting in the production of H_2_ and carbon‐based fuel production from PET and PLA polymers under aqueous alkaline conditions at room temperature (Figure 5d,e).^[^ ^53^ ^]^ This approach not only represents a mild, simple, and low‐energy strategy for the conversion of plastic waste into valuable chemical products, but also avoids the use of toxic Cd‐based photocatalysts under visible light conditions.^[^ ^121^ ^]^ This is likely to be an effective approach for tackling the issue of recycling waste plastics.

It is well known that the nanosizing catalysts can significantly improve their catalytic capacity.^[^ ^124^, ^125^ ^]^ However, the aggregation of nanocatalysts during the catalytic process can lead to problems such as difficult exposure of catalytic active sites and reduced catalytic capacity. To overcome this issue, researchers have proposed the in situ conversion of unstable metal sites to nanoparticles with semiconducting properties on a bimetallic metal–organic framework (MOF) to prevent nanoparticle aggregation during photocatalysis. FeAg‐MOF was selected as the precursor for the preparation of Ag_2_O/Fe‐MOF photocatalysts for the degradation of PE, PEG, and PET for H_2_ generation.^[^ ^126^ ^]^ Ag_2_O/Fe‐MOF has a wide light collection range and an abundance of active sites, making it easier to upgrade plastics into value‐added chemicals together with H_2_ production. This provides ideas for the design of advanced heterojunction photocatalysts and the conversion of waste plastic into high‐value‐added products and clean hydrogen energy. It is understandable that the photocatalytically generated holes and electrons can act on different substances to produce different substances. The formation of heterojunctions makes this result more obvious. The photoreforming system built with MoS_2_/CdS as heterostructure has successfully achieved hydrogen precipitation in the treatment of PLA, PET, and PE plastic waste.^[^ ^127^ ^]^ This system has been used for the production of high‐value‐added carboxylates (e.g., formates, acetate, and ethanoates) and gaseous alkanes (e.g., methane, ethane, propane, and pentane) at the same time, facilitating the upcycling of waste plastics in an environmentally friendly and economically viable manner. Therefore, multicomponent catalysts are considered to be an important direction for future sustainable development, with promising prospects for their application in the field of waste plastic upcycling.

To avoid excessive oxidation of plastics during upcycling process, in addition to the forementioned surface coating and the selection of photocatalysts with narrow intrinsic bandgaps, another alternative approach involves regulating the bandgap of photocatalysts through heteroatom doping or heterostructure building in a rational way. This approach could effectively promote charge transfer and inhibit the complexation of e^−^ and h^+^, significantly improving the activity and stability of photocatalysts and enabling the upcycling of photocatalytic plastics.

**Figure 6:**
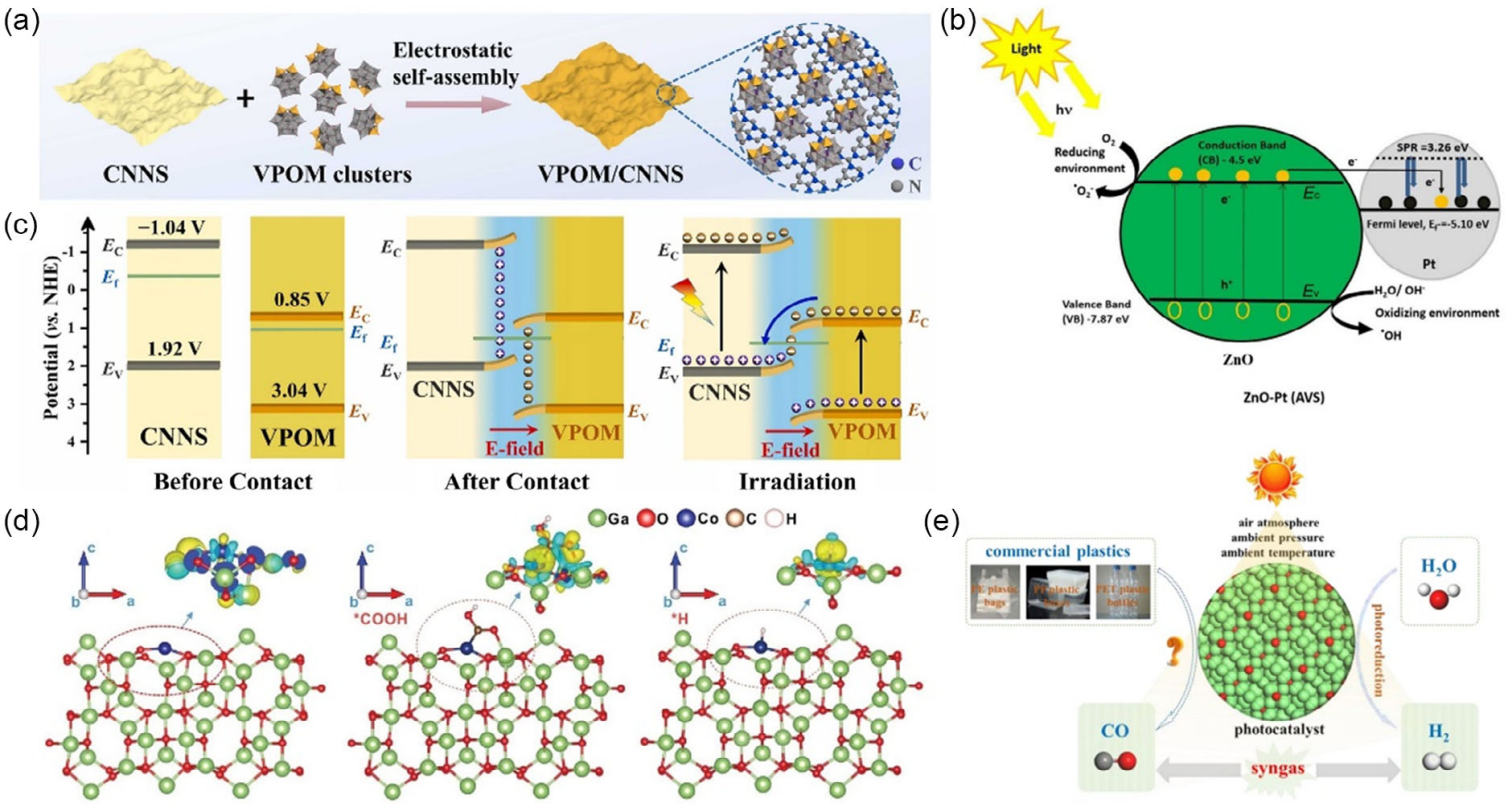
a) Scheme of VPOM/CNNS heterojunction photocatalyst prepared though self‐assembly process.^[^ ^129^ ^]^ Copyright 2022, Elsevier Ltd. b) Proposed mechanism of enhanced photocatalytic activities with ZnO–Pt catalyst.^[^ ^130^ ^]^ Copyright 2019, MDPI. c) Energy band diagram of VPOM/CNNS Z‐scheme heterojunction catalyst.^[^ ^129^ ^]^ Copyright 2022, Elsevier Ltd. d) Investigation of Co atoms in the Co–Ga_2_O_3_ nanosheets for the plastic‐to‐syngas process. e) Diagram of plastics‐to‐syngas photocatalyzed using Co‐doped Ga_2_O_3_ (Co‐Ga_2_O_3_) nanosheets.^[^ ^132^ ^]^ Copyright 2022, Oxford Univ Press.

Formic acid is also considered to be a chemical of wide research value as an excellent storage medium for hydrogen.^[^ ^128^ ^]^ Self‐assembled V‐substituted phosphomolybdate clusters/g‐CN_*x*_ nanosheets (VPOM/CNNS) have been designed and prepared to create Z‐type heterostructures for upcycling PE, PP, and PVC to produce formic acid using visible light (**Figure** **6**a,c). Transient absorption spectroscopy and ESR have demonstrated that this structure not only has Z‐type charge transfer but also has high charge separation efficiency and redox potential. The upcycling yield of this structure for PE was shown to be 262 times higher than that of the original CNNS.^[^ ^129^ ^]^ It was elucidated that the construction of heterojunction could significantly enhance the catalytic ability of the catalyst for the degradation of waste plastics with stable C–C backbone as the main chain.

In addition to the use of two appropriate semiconductors for matching bonding on the bandgap, heterojunction catalyst materials could be constructed by combining semiconductors with metal that have significant plasmon resonance effects. For example, the plasmonic effect of Pt promotes better separation of excitons at the semiconductor interface, which in turn increases the hydroxyl radical activity and increases the light absorption in the visible region by 78%. This is due to the diffusion of photogenerated electrons from the ZnO nanorod interface into Pt, which reduces the recombination of h^+^/e^−^ pairs and thus enables effective degradation of low‐density polyethylene (LDPE) plastic chips (Figure 6b).^[^ ^130^ ^]^ Although, the plastics degradation ability of semiconductor catalysts could be enhanced *via* noble metals to some extent by increasing the absorption of light, our emphasis is not only on degradation but also on resourceful upcycling. For example, after simple treatment with ammonia solution, PLA can be upconverted to alanine using Ru/TiO_2_ as catalyst.^[^ ^131^ ^]^ This not only transforms the waste plastic into high‐value‐added chemicals, but also effectively inhibits the carbon‐emitting process.

Besides the design and preparation of heterojunctions for photocatalysts, heteroatom doping is also an effective pathway to regulate the catalytic ability of photocatalysts. For example, Co–Ga_2_O_3_ nanosheets were used as catalysts for the degradation of various postconsumer plastics including PE bags, PP boxes, and PET bottles, ultimately generating synthetic gases such as H_2_ and CO (Figure 6d,e).^[^ ^132^ ^]^ Obviously, the heteroatom doping significantly changes the intrinsic photoresponsive properties of the original semiconductor material, which can be utilized to control the original properties of the semiconductor and develop new photocatalysts.^[^ ^133^ ^]^ Moreover, the size and morphology of the metal present in the heterogeneous catalysts play a critical role in the activity, selectivity, and stability of the material.

#### 2.2.3 Single‐Atom Catalyst

The quest for catalysts with improved activity, enhanced selectivity, and clear structure–activity relationships continues despite the significant advancements of nanocatalysts in recent decades. Single‐atom catalysts are a novel type of catalyst in which metal atoms are dispersed in a support, distinguishing them from nanoparticle catalysts. These catalysts provide 100% utilization of metal atoms and maximal metal–support interfaces, resulting in unique catalytic behavior.^[^ ^134^ ^]^ For example, integrated cobalt single‐atom catalysts are used for glycolysis of polyesters due to their strong photothermal conversion, high catalytic activity, and stability. This enhances nucleophilic addition elimination processes, resulting in much higher space–time yields compared to general catalysts. Photothermal catalysis of polyethylene terephthalate (PET) has conversion and yield rates that are 5.4 and 6.6 times higher than thermal catalysis respectively. Although single‐atom catalysts have emerged as a promising field for the catalysis of small‐molecule conversion, the area of single‐atom catalysis for plastic recycling remains largely underexplored. There are mainly the following scientific and engineering problems that account for this. First, the photocatalytic degradation process is more complex than small‐molecule catalytic transformation, posing a challenge for single‐atom catalysts to meet the requirements. Second, single‐atom catalysts are limited to specific light wavelengths, rendering them ineffective under varied light conditions. Finally, their poor stability and limited ability to sustain catalytic effects make them impractical for industrial purposes.

Despite the aforementioned issues, given its outstanding catalytic effect and promising research outcomes in the catalytic conversion of small molecules, there is a need to further investigate its potential in the realm of polymer degradation. Single‐atom catalysts, relying on Earth‐abundant metals, must be designed to enhance the sustainability and economic feasibility of the nanocatalytic chemical upcycling of plastic waste. To achieve this objective, these catalysts should exhibit improved conversion efficiency, product selectivity, substrate versatility, and reusability.

Since the light‐driven catalytic degradation process requires a long time to achieve complete degradation of postconsumer waste plastics, this upcycling approach is not presently suitable for industrial applications. Nonetheless, considering the low‐energy consumption, low CO_2_ emission, and the fact that upcycling is still in the initial stage of research, photocatalytic plastics upgrading remains a promising prospect. Furthermore, both photochemical oxidation and photocatalytic oxidative degradation technologies are based on the injection of energy carried by high‐energy photons to trigger the generation of free radicals, leading to the breaking of polymer chains and achieving degradation. In particular, photocatalytic oxidation technology is basically based on the degradation of polymers by irradiating a semiconductor material with s large bandgap by a short‐wavelength and high‐power light beam, resulting in the generation of highly reactive free radical species.^[^ ^135^ ^]^ Although photocatalysis achieves substantially improved degradation efficiency compared to photochemical oxidative degradation, the stringent requirements for excitation light also make the degradation of MPs by light somewhat limited, and the development of catalysts with excellent visible light response presents an opportunity to address waste plastics. Additionally, the upcycling of current waste plastic typically generates a range of carbon oxides and nitrogen oxides, and selectively converting it to a single value‐added product remains challenging (**Table** **1**).

**Table 1:** Different productions from waste plastics via photocatalysis process

Type of plastics	Catalytic	Conditions	Products	Refs.
LDPE	polyacrylamide grafted TiO_2_	UV light	CO_2_	[182]
PET, PLA	CN_*x*_/NiP	Solar light	H_2_	[53]
PP	TiO_2_–rGO	Solar light	Fragments	[117]
PET	CdS	Visible light	H_2_	[121]
LDPE	ZnO	UV light	CO_2_	[183]
PP	TiO_2_ NPs	UV light	CO_2_; H_2_O	[184]
PS	Mo–TiO_2_; W–TiO_2_	UV light	/	[133]
PE, PEG, PET	Ag_2_O–Fe–MOF	Solar light	H_2_, CH_3_COOH	[126]

### 2.3 Photothermal Catalytic Degradation

Since most of the research works in photocatalysis plastics upcycling have focused on the developing efficient catalysts in mild environments, it is still difficult to activate and cleave of chemical bonds requiring dissociation energies.^[^ ^136^ ^]^ Therefore, a catalytic process using coupled light and heat for the degradation of waste plastics has recently emerged. This photothermal catalytic process combines the advantages of photocatalysis and thermocatalysis, significantly improving the chances of C—C bond cleavage.^[^ ^137^ ^]^ For example, the combined effect of low‐quality infrared light and visible light can achieve heating of carbon‐based materials,^[^ ^138^ ^]^ increasing the thermal vibration of the reaction substrate and further increasing the rate of the chemical reaction. This injection of thermal energy is not limited to the heating of the reaction substrate, but also includes the heating of the catalyst material. Thus, the photothermal catalysis process aims to increase the reaction rate to a new level by combining the advantages of both photocatalysis and thermocatalysis. We know that materials with photothermal conversion capability, which also possess photocatalytic capability, will provide a more solid feasibility for photothermal catalysis. In summary, the photothermal effect could be divided into three forms, including local plasma resonance, nonradiative relaxation processes in semiconductors after light absorption, and thermal vibrations of chemical bonds in molecules.^[^ ^139^, ^140^, ^141^ ^]^

Photothermal cocatalytic processes could be broadly divided into three strategies. The first strategy is thermally assisted photocatalysis, in which externally injected thermal energy is used to reduce the reaction activation energy of the feedstock, while minimizing its effect on the catalyst. At the time, the thermal energy could also accelerate the mass transfer efficiency in the reaction system, which gives the reactants and the catalyst a greater chance of collision and reaction.^[^ ^142^ ^]^ The second strategy is light‐assisted thermal catalysis, which operates based on the photothermal conversion effect. Light energy produces a local temperature increase based on thermal catalysis^[^ ^139^ ^]^ and thus promotes the local high‐temperature catalysis of the reaction substrate on the catalyst surface.^[^ ^122^ ^]^ Particularly in cases where the catalyst has nanostructure with tips,^[^ ^143^ ^]^ narrow channels,^[^ ^144^ ^]^ and confined spaces,^[^ ^47^ ^]^ the temperature of the catalyst at the corresponding position could be significantly increased under light illumination. The third strategy is photothermal coupled catalysis, which involves the mutual promotion of photocatalytic and thermal catalytic reactions. After absorbing light energy, the catalyst is used to generate thermal energy for reducing the activation energy of the surface substrate, in addition to the separation of h^+^/e^−^ pairs to produce the photocatalytic reaction.^[^ ^145^ ^]^ In addition, photocatalytic reactions can also improve catalytic efficiency while accelerating the reaction rate through conventional thermal catalysis.^[^ ^146^ ^]^

**Figure 7:**
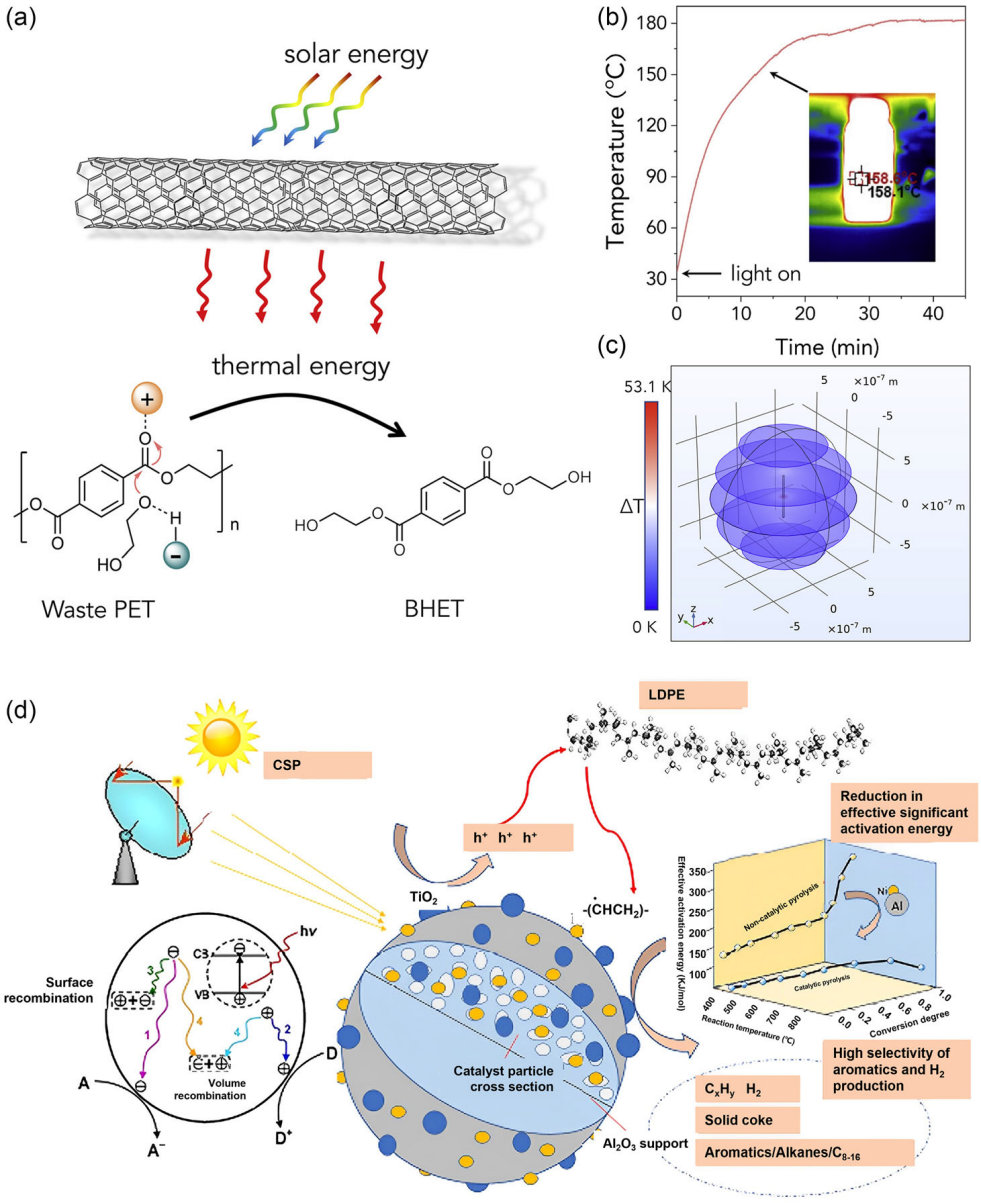
a) Scheme of photothermal conversion for PET degradation. b) Temperature curve of the EG solution containing 0.5 wt% of CNT‐PDA shone with simulated sunlight. The inset shows the thermal image. c) Simulated temperature gradient field around one CNT‐PDA photothermal conversion material.^[^ ^148^ ^]^ Copyright 2022, Cell Press. d) Mechanism for photothermal catalysis and its effects on the gas products of LDPE catalysis.^[^ ^150^ ^]^ Copyright 2022, Elsevier Ltd.

As previously mentioned, the confined space formed by the core–shell structure not only improves the stability of the catalyst, but also helps to prevent sintering and scorching of the material in the central region during annealing.^[^ ^147^ ^]^ The SiO_2_ sheath provides thermal insulation and infrared shielding, limiting the photothermal energy dissipation of the Ni core during illumination, ultimately resulting in a super photothermal effect that improves the photocatalytic capabilities of the Ni core. Materials with high photothermal conversion capabilities are highly promising for a wide range of light‐assisted thermal catalysis applications, particularly in the upcycling of plastics. For example, local heating during photothermal catalytic degradation of PET has been achieved by poly(dopamine) pre‐modified multiwalled CNTs (CNT‐PDA) (**Figure** **7**a–c).^[^ ^148^ ^]^ In just 30 minutes, the temperature at which dispersion occurs was raised to ≈180 °C by adding 0.5 weight percent of solar absorbers to ethylene glycol (Figure 7b). Moreover, a temperature gradient can be observed between the solar absorber and the surrounding area, which is absent in the thermal catalysis system (Figure 7c). In the meantime, this approach has yielded a degradation efficiency 3 times higher than thermal catalysis at a lower‐temperature profile of only 150 °C for plastics upcycling. In addition, the high penetrating power of near‐infrared (NIR) light is advantageous for heterogeneous catalytic processes, as it can directly activate the internal molecules.^[^ ^149^ ^]^ Therefore, catalytic materials with NIR light absorption capability will be very useful in the photothermal catalysis of plastics. The development and preparation of new catalysts is a key focus of chemist research, and Ni‐TiO_2_‐γ‐Al_2_O_3_ bifunctional photothermal catalysis has been developed for resourceful upcycling and recovery of LDPE (Figure 7d).^[^ ^150^ ^]^ The catalyst, used in a fixed‐bed reactor heated by a 500 °C solar simulator, has shown success in producing hydrogen and aviation fuel liquid products, including olefins, aromatics, etc. The gratifying results of highly stable plastic upcycling are attributed to the relaxation of electrons generated within and between the bands of photoexcited plasma metal Ni, as well as the exceptional carrier transfer capability of the nonplasma semiconductor TiO_2_. Additionally, the photothermal catalytic cracking of LDPE was similarly simulated with sunlight. Photothermal catalysis with Ni/TiO_2_/Al_2_O_3_ as the catalyst regulating the production process can produce CNTs with a yield of 280 mg g^−1^ plastics, along with a H_2_ yield of 54 mmol g^−1^ plastics.^[^ ^46^ ^]^ This was achieved by mediating the metal–carrier interaction in NiTiAl through photoelectron transfer to facilitate the growth and hydrogen precipitation of CNTs. Although photothermal catalysis has the characteristics of relatively difficult implementation and poor economic practicability due to high‐energy density input, it is viewed as an efficient catalytic technology.

## 3 Electroupcycling Platform

Similar to photocatalysis, the electrochemical method has received extensive attention in many fields, including water splitting,^[^ ^151^ ^]^ carbon dioxide reduction,^[^ ^152^ ^]^ organic synthesis,^[^ ^153^ ^]^ biomass degradation,^[^ ^154^ ^]^ etc. Therefore, electrochemical technology is considered as a more stable and controllable potential technology in waste plastics upcycling.^[^ ^155^ ^]^ If the electrical energy used is obtained from renewable sources, electrocatalytic degradation of plastics for hydrogen production and upcycling into high‐value‐added small‐molecule compounds would be an effective way to achieve sustainable development. Electrochemical catalysis is a process in which electrical energy serves as an external excitation source to facilitate the chemical transformation of substrates.^[^ ^156^ ^]^ This process is divided into two types, anodic oxidation catalysis and indirect cathodic oxidation catalysis. In anodic oxidation catalysis, the target substrate undergoes direct oxidation on the anode surface by charge transfer. On the other hand, indirect cathodic oxidation catalysis involves the oxidation of the target material by HO^•^ or other oxidizing substances (active chlorine, H_2_O_2_, O_3_ and persulfate, etc.) produced in aqueous solution. The overall degradation performance would be largely determined by the intrinsic properties of the electrode materials in the electrochemical catalytic system. Therefore, the selection of efficient catalyst materials is extremely critical. Numerous studies have reported the possibility of converting byproducts into chemical feedstocks by electrochemical techniques.^[^ ^157^ ^]^ However, there are relatively few reports on the application of electrochemical catalysis for the degradation of polymeric plastics.

Compared to thermo‐ and photocatalytic degradation technologies, electrocatalysis has several similar advantages. For example, the electrocatalytic technology can increase the economic value of the products and reduce environmental pollution when the power for redox electron transfer is replaced by renewable technologies (e.g., wind and photovoltaic).^[^ ^158^ ^]^ Electrocatalysis could be carried out at moderate temperatures and atmospheric pressures, avoiding the need to overcome the reaction barriers arising from activation energy in reaction kinetics through harsh conditions such as high temperatures and pressures.^[^ ^159^ ^]^ Additionally, the electroupcycling platform has some unique advantages. For instance, electrocatalysis may have the capacity to improve the selectivity of the desired high‐value‐added products to a certain extent by modulating the electrochemical driving potential.^[^ ^160^ ^]^ Electrocatalysis, where the electrode acts as a heterogeneous catalyst in the electrochemical process, can overcome the challenges of product–catalyst separation and further improve the products purity.^[^ ^161^ ^]^ Furthermore, the electroreforming strategy enables the conversion of intermittent and fluctuating renewable electrical energy into chemical energy, which can be conveniently stored and transported.^[^ ^162^ ^]^ Hence, the electrocatalytic technology offers an effective way to convert plastics, which not only helps combat pollution but also enhances the value of waste materials.

The electrocatalytic process requires an electric current to form a circuit, so the type of conductive medium, the level of applied bias, the heating or not, and the choice of catalyst in this system can significantly affect the selectivity of the final products. In general, electrocatalytic degradation takes place in aqueous solutions at ambient temperature and pressure and is considered as an alternative route to thermal degradation of polymers. Studies have been conducted using electrocatalysis to convert biomaterials such as lignin into chemical feedstocks.^[^ ^163^ ^]^ However, there are few studies on the electrocatalytic conversion of plastic waste, which may be related to the high hydrophobicity and poor dispersibility of most plastics in aqueous solutions.

### 3.1 Electrocatalytic Degradation

#### 3.1.1 Single‐Component Catalysts

In general, the oxidation of small‐molecule compounds is easily achieved in alkaline aqueous solutions. For instance, a study has reported the use of mesoporous SnO_2_ grown on carbon cloth (mSnO_2_/CC) and CuO nanosheets grown on copper foam (CuO NS/CF) as cathodic and anodic electrocatalysts for the upcycling of plastics.^[^ ^51^ ^]^ Here, the oxidation of methanol by the anode and the reduction of carbon dioxide by the cathode could be used for the efficient production of formic acid simultaneously. In addition, the anodic and cathodic oxidation of glycerol and reduction of carbon dioxide in the presence of Co‐based catalysts enabled the synthesis of a series of C1 and C2 products, respectively.^[^ ^157^ ^]^ Therefore, the electrocatalytic reduction of CO_2_, a fully mineralized product of plastics, could be fully achieved by modulating the preparation of electrode catalysts under conventional strong alkaline conditions and combining the preparation of high‐value‐added products. Since different reactions occur at the anode and cathode in the electrolytic cell, the anode and cathode of the electrolytic cell could be isolated by a glass frit double‐chamber reactor (commonly known as the H‐cell) in order to accurately study the reaction processes occurring at the different electrodes and to improve the products’ purity. Recently, it has been reported that the use of high concentrations of corrosive NaOH solutions and the further reaction of the product terephthalic acid can be avoided in NaCl solutions of methanol and water conditions when depolymerizing PET plastics.^[^ ^164^ ^]^ The search and exploration of new solvents for electrocatalytic degradation instead of strong alkaline environments will be a very challenging research task.

**Figure 8:**
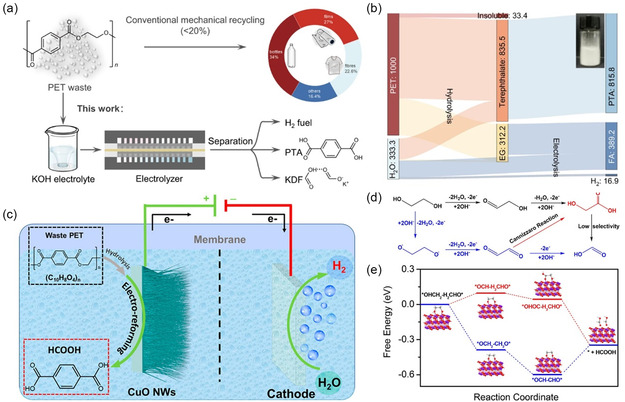
a) Electrocatalytic conventional route for PET upcycling. b) Sankey diagram for the mass flow of PET upcycling. The inset shows the photograph of separated high‐purity PTA.^[^ ^52^ ^]^ Copyright 2021, Nature Portfolio. c) Scheme of hybrid electrolysis for electroreforming of the PET. d) Proposed reaction route for the oxidation of EG to formic acid. e) Reaction free energy diagram of EG oxidation on CuO by different pathways.^[^ ^167^ ^]^ Copyright 2022, American Chemical Society.

Postconsumer plastics should be considered as mass raw material resource to be exploited for making chemicals and fuels, rather than as waste that poses environmental and biological threats.^[^ ^165^ ^]^ The electrocatalytic conversion of small molecules suggests that the electrocatalytic upcycling of plastics will present us with new opportunities and challenges. The electrocatalytic CO_2_ reduction reaction can act as part of the tandem reaction to reduce the CO_2_ generated after the complete mineralization of plastics into high‐value‐added small‐molecules chemicals Therefore, the tandem reaction would be an excellent solution to the problem of CO_2_ generation during the degradation of plastics. Electrocatalytic upgrading of PET plastics using a nickel‐modified cobalt phosphide (CoNi_0.25_P) electrocatalyst by tandem hydrolysis of PET and electrocatalytic oxidation of hydrogen evolution reaction (HER) and EG enables the production of valuable commercial chemicals (potassium dicarboxylate and terephthalic acid) and H_2_ fuel (**Figure** **8**a,b).^[^ ^52^ ^]^ This process with economic practicality has an 80% Faraday efficiency and formic acid selectivity, providing an experimental basis for the conversion of waste plastics into treasure, and generates long‐term economic benefits.

#### 3.1.2 Multicomponent Catalysts

In the realm of electrocatalytic degradation of polymers, multicomponent catalysts also offer notable advantages compared to single‐component catalysts. These advantages emerge from the synergistic interactions of the different components within the multicomponent system, which result in enhanced catalytic efficiency. Likewise, the various components of these catalysts can counteract or share the poisoning effect of the reaction, leading to improved stability and antitoxicity. The multicomponent nature of the catalysts also allows for greater regulation of activity, which can be tailored to different reaction conditions and catalytic reaction pathways. Additionally, improved catalyst utilization rates can reduce the amount of catalyst required and resultantly lower costs. Given these extensive benefits of multicomponent catalysts in polymer degradation, further research and analysis on the subject is imperative.

An excellent research study highlights that Pd‐modified Ni foam catalysts upgrade waste PET to high‐value‐added chemicals. These chemicals include terephthalic acid glycol esters, carbonates, and H_2_ fuel, produced at the anodic and cathodic ends.^[^ ^166^ ^]^ At the same time, the catalyst shows high selectivity (95%) and high Faraday efficiency (93%) for the product carbonic acid. Similarly, CuO nanowires (NWs) catalysts could selectively catalyze the conversion of EG to formate products (Figure 8c,d), and the density flooding theory (DFT) calculations indicate that EG is oxidized to glyoxal intermediates on CuO NWs and then further cleaved by C—C bonds to formic acid (Figure 8e).^[^ ^167^ ^]^ These findings emphasize the crucial role of catalyst design and preparation in improving catalytic performance and selectivity in both photocatalytic and electrocatalytic degradation processes. NiCo_2_O_4_, a binary metal oxide electrocatalyst, has the ability to produce formic acid at both cathode and anode ends, resulting in the electrocatalytic mineralization of PET plastics coupled with CO_2_ reduction. This methodology offers advantages such as production of value‐added chemicals and reducing environmental pollution.^[^ ^168^ ^]^ Moreover, the Faraday efficiency can reach as high as 155% in the production of formic acid at a cell voltage of 1.90 V. We believe that binary, multimetal oxide, and high‐entropy materials have the opportunity to be excellent electrocatalytic materials for the degradation of plastics and CO_2_ reduction.

Indeed, the goal of laboratory exploration and experimentation not only holds the potential to achieve practical applications in industry but also has the ability to promote the advancement of scientific knowledge. Ni_3_N/W_5_N_4_ electrodes have Pt‐like HER performance and excellent industrial current stability (≈300 h), thanks to the synergistic effect of barrier‐free heterogeneous interfaces, superhydrophilic surfaces, and multistage Janus structures.^[^ ^169^ ^]^ It's worth noting that this work also highlights the efficient production of H_2_ and HCOOH using Ni_3_N/W_5_N_4_ electrocatalysts in plastic‐filled seawater driven by photovoltaic power. This has promising potential for industrialization efforts.

The electrocatalytic and photocatalytic degradation of polymers share similarities in that they both utilize various types of catalysts, such as single‐component, multicomponent, heterojunction, and single‐atom catalysts. For instance, the Ru catalyst supported by CeO_2_ is an exemplary single‐atom catalyst that inhibits the methanation process, preventing the terminal C–C cleavage and fragmentation in the polyolefin chain. This achievement addresses the economic challenge of catalytic hydrogenolysis in waste polyolefin recycling, promoting plastic waste reduction and environmental remediation. Nonetheless, the current research content regarding electrocatalytic degradation of polymers is relatively limited and will not be elaborated here.

### 3.2 Electro‐Assisted Degradation

#### 3.2.1 External Factors‐Assistive Technology

**Figure 9:**
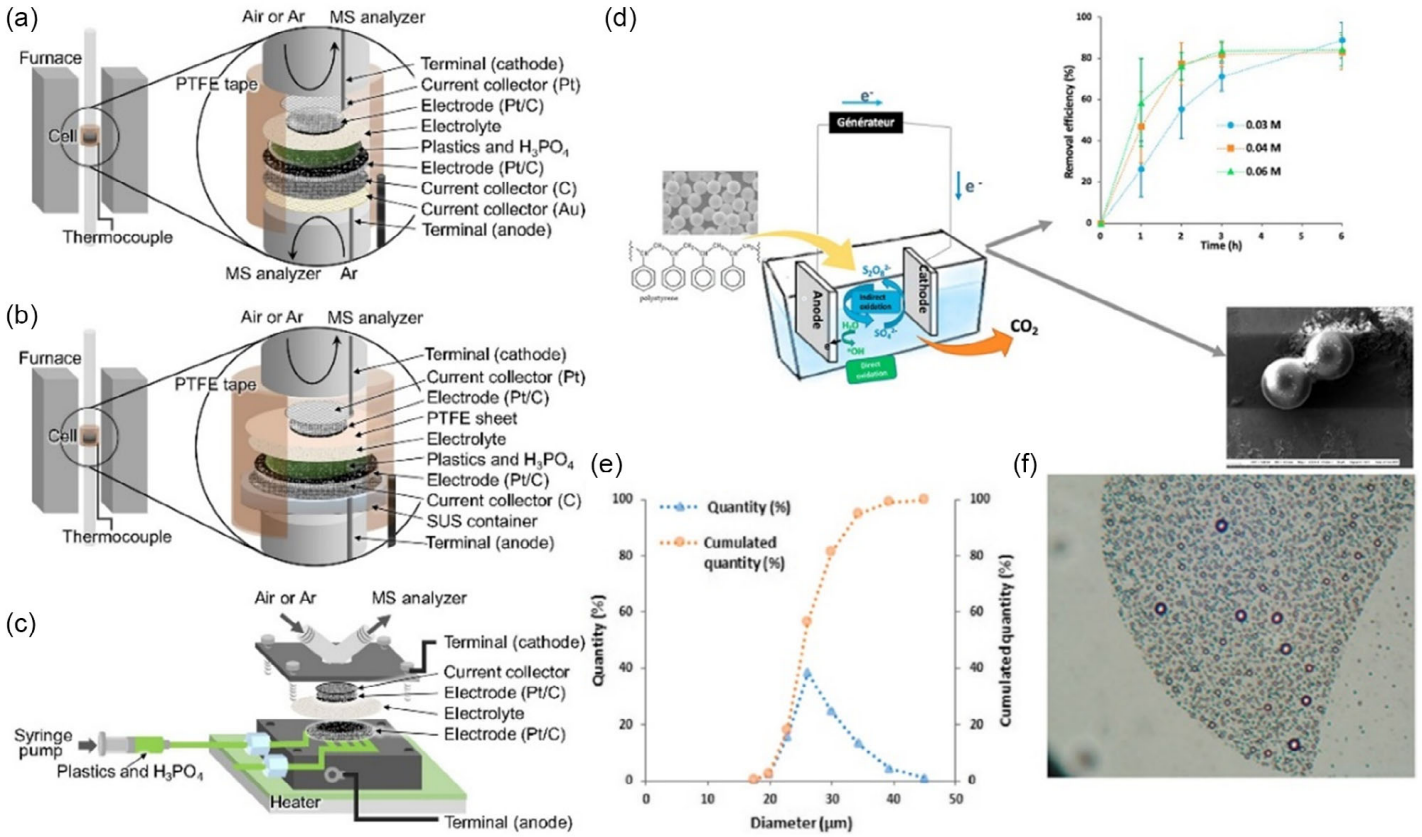
a) Illustrations of three types electrochemical fuel cells: the a) two chamber, b) batch, and c) flow cells.^[^ ^170^ ^]^ Copyright 2020, Elsevier Science Ltd. d) Schematic diagram of electrocatalysis reaction for PS MPs. e) Size distribution of the used PS MPs. f) The view of PS MPs under an optical microscope.^[^ ^173^ ^]^ Copyright 2021, Elsevier Ltd.

**Figure 10:**
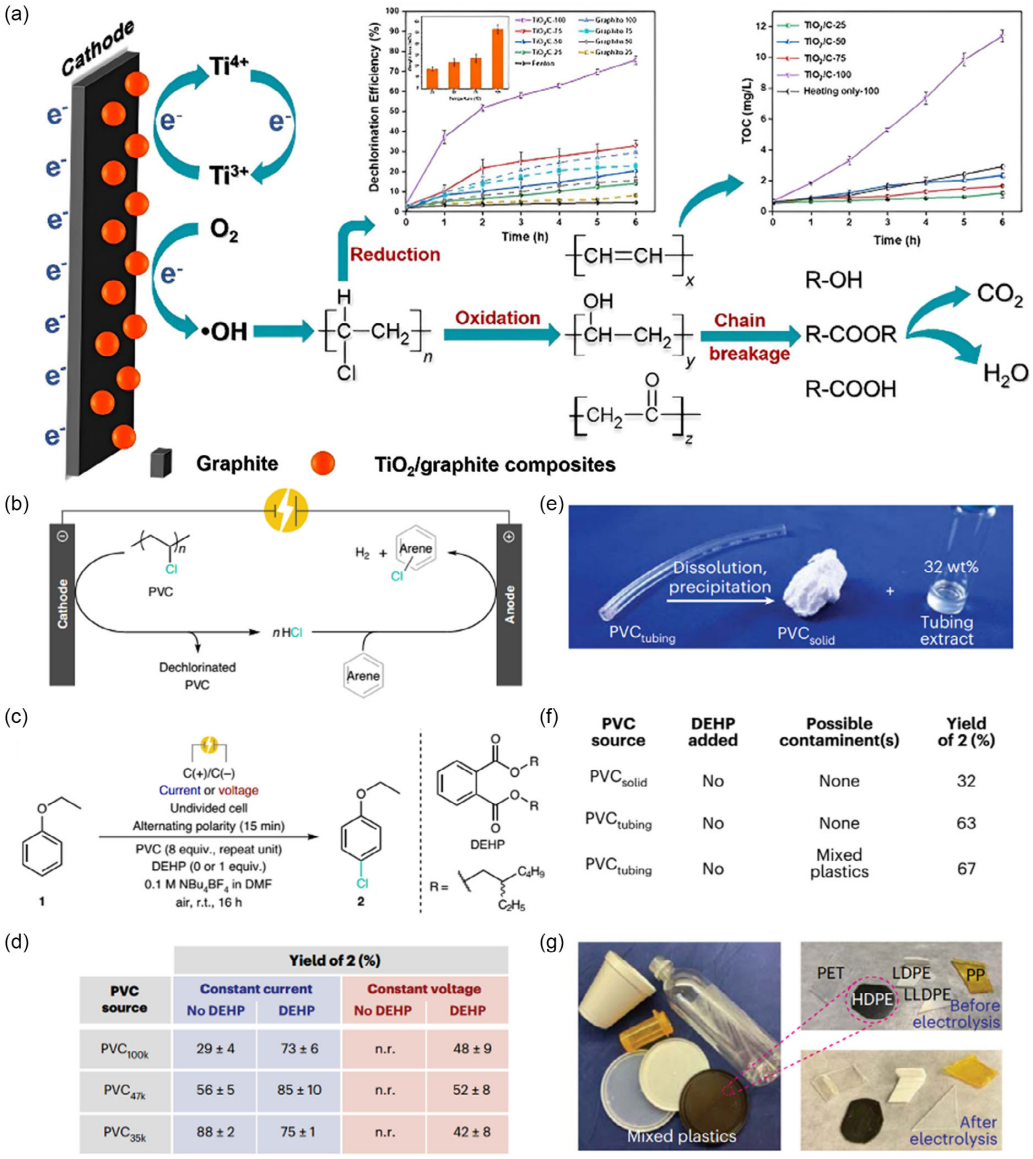
a) Degradation of PVC MPs via an EF‐like system and relative results.^[^ ^171^ ^]^ Copyright 2020, Elsevier Ltd. b) Scheme for reductive dichlorination of PVC (cathode) and oxidative chlorination of an arene (anode) via paired electrolysis reaction. c) The model reaction and relative conditions evaluated in which ethoxybenzene (1) is chlorinated using PVC and DEHP. d) The results show the higher yields with PVC of lower‐molar mass during the chlorination reaction procedure. e) PVC tubing utilized to simulate PVC waste in the paired electrolysis reaction. f) Yields of PVC plastic with various conditions under the charging of the galvanostatic reaction using arene 1 as the chlorination reagent. g) Plastic items used to simulate the mixed‐plastics‐waste stream in real life.^[^ ^174^ ^]^ Copyright 2023, Nature Portfolio.

Electrocatalytic plastic upcycling could be considered as an effective alternative for thermal degradation, but it is undeniable that the participation of heat significantly increases the efficiency of electrocatalysis. For example, the conversion of polyvinyl alcohol (PVA) to H_2_ (9.5 mol min^−1^) in a solution of H_3_PO_4_ at 200 °C could be achieved successfully by applying only mild external voltage (0.55 V) (**Figure** **9**a–c).^[^ ^170^ ^]^ In addition, electrocatalytic upcycling of PVC was carried out on TiO_2_/C cathode (−0.7 V) in a 100 °C environment to form carboxylic acid (75%) (**Figure** **10**a).^[^ ^171^ ^]^ This approach could produce a high purity of single product that can be used directly as fuel without post‐treatment. The organic combination of thermal and electrocatalytic degradation can greatly facilitate the production of chemical products compared to the use of electrocatalytic degradation alone. The conversion of PP plastics to H_2_ and C1–C5 fuels could be achieved by solar heat generation coupled with electrocatalytic degradation. Elevated photothermal temperature (350–390 °C) and electrolysis current (0–400 mA) in the system significantly enhance the ability to depolymerize PP plastics.^[^ ^172^ ^]^ Certainly, in addition to increasing heat, modulating the voltage can also achieve the degradation of plastics, with the anodic oxidation method capable of degrading PS within 1 h. MPs do not decompose into smaller particles, but directly degrade into gaseous products (Figure 9d–f).^[^ ^173^ ^]^ However, the large energy consumption is a major obstacle on the way to real industrial application. Therefore, it is promising to achieve an efficient electrocatalytic degradation system at low temperature and low current. Although PVC could be degraded by electro(de)chlorination pathway and used for the synthesis of chloroarenes in a paired electrolysis reaction without catalyst,^[^ ^174^ ^]^ we still need to focus our efforts on the development of new high‐performance catalysts (Figure 10b–g).

#### 3.2.2 Electro‐Fenton Technology

Another strategy to potentially increase the conversion efficiency of electrocatalytic degradation systems is the use of the Fenton chemistry often referred to as the EF process. The EF technology is dominated by the indirect oxidation of the cathode in the electrocatalytic system.^[^ ^72^ ^]^ The Fenton reaction in general is the homogeneous cleavage of H_2_O_2_ mediated by several transition metals (Fe, Co, Mn, etc.) and generating HO^•^ active species, as shown in Equation (4).^[^ ^175^ ^]^ The free radical is highly reactive, which allows it to mineralize most organic matters to carbon dioxide and water, while also causing other cascade reactions.^[^ ^176^ ^]^ In contrast, the electrocatalytic process provides a highly efficient and controllable pathway for the two‐electron reduction and oxidation of O_2_ and water, respectively, via Equation (5) and (6).^[^ ^175^ ^]^ Due to the high controllability, ease of operation, and low secondary pollution, EF reaction technology has been successfully applied to degrade many organic pollutants.^[^ ^177^ ^]^ Therefore, we believe that EF technology will be used as a more energy‐efficient method for upcycling a wider range of waste plastics.

(4) $$H_{2}O_{2}+\text{Fe}\left(\text{II}\right)\to \text{Fe}\left(\text{III}\right)+{\text{OH}}^{-}+{\text{HO}}^{•}$$

(5) $$O_{2}+\;2H^{+}+2e^{-}\to H_{2}O_{2}$$

(6) $$2H_{2}O\to H_{2}O_{2}+\;2H^{+}+2e^{-}$$

(7) $$\text{Fe}\left(\text{III}\right)+e^{-}\to \text{Fe}\left(\text{II}\right)$$

For example, an EF‐like system with TiO_2_/graphite as cathode achieved the degradation of PVC through the large amount of HO^•^ produced in the system. This showed outstanding activity in the oxidative and cathodic dichlorination process of PVC and also obtained a variety of useful chemical products during the degradation procedure.^[^ ^171^ ^]^ Although this work was only achieved the degradation of PVC plastics, it remains unclear whether the system is applicable to the degradation of other types of plastics. Nevertheless, this method still provides a research direction to explore an efficient electrochemical oxidation way to degrade plastics into small‐molecule products.

### 3.3 Synergistic Photoelectro Upcycling

**Figure 11:**
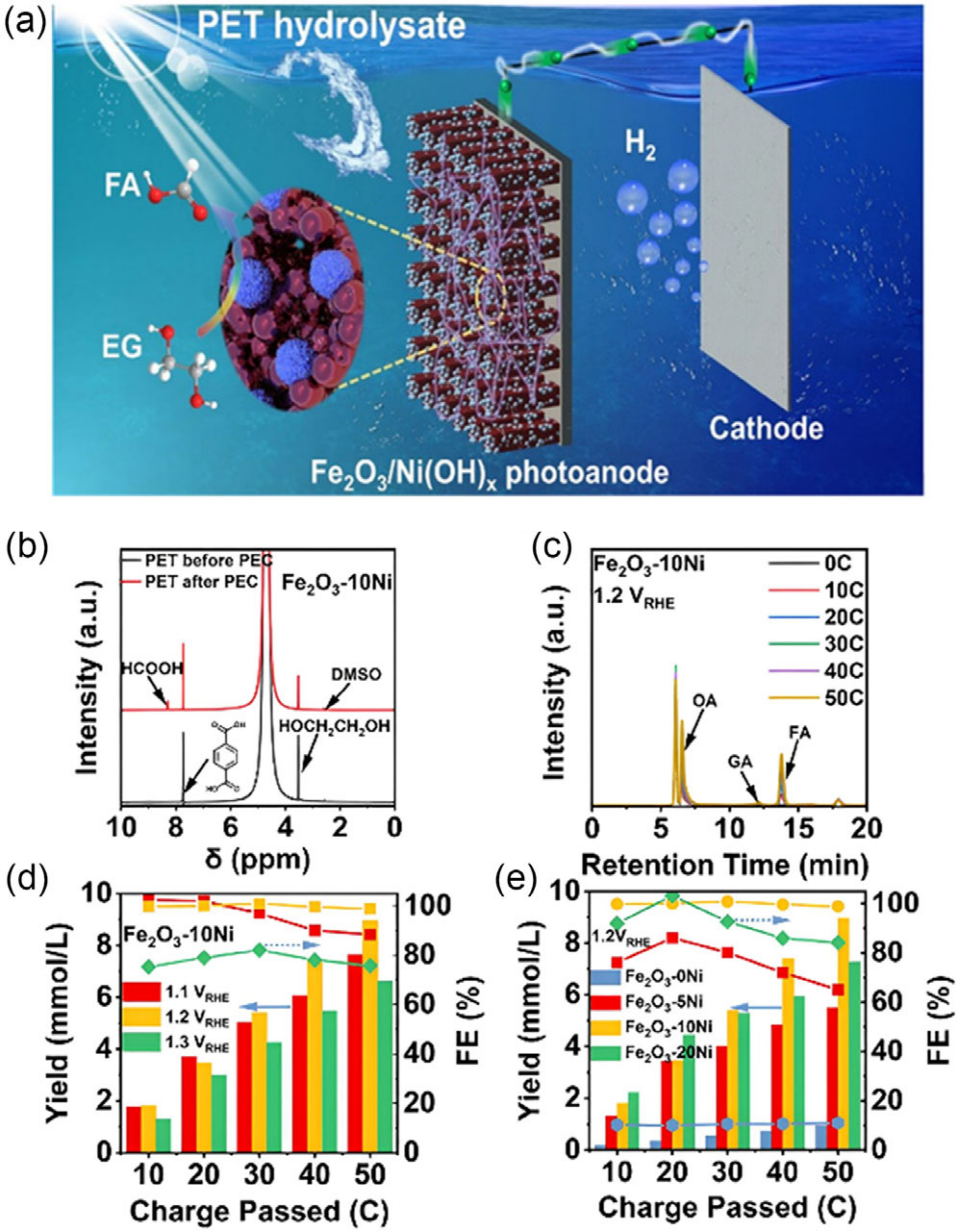
a) Schematic diagram of photoelectrochemical catalysis of PET plastic to coproduce formic acid and H_2_. Results of b) ^1^H NMR and c) high performance liquid chromatography (HPLC) determination of the oxidation products of the PET degradation via PEC valorization. d) Yields of the generated FA and the corresponding FE of Fe_2_O_3_–10Ni catalyst at different applied and e) different samples at the applied bias of 1.2 V_RHE_ for Fe_2_O_3_–xNi photoelectrodes.^[^ ^179^ ^]^ Copyright 2022, American Chemical Society.

As mentioned previously, the coupling of electrocatalysis and thermal catalysis can greatly increase the reaction rate to a great extent, so the coupling of electrocatalysis and photocatalysis has also gradually attracted much attention. This approach has been successfully applied in the overall water splitting and reforming of EG and PET plastics through a nanonickel phosphorus alloy (nano Ni–P) precatalyst.^[^ ^178^ ^]^ Nano Ni–P not only serves as an ideal catalyst for OER, but also selectively converts EG and PET to formic acid. More importantly, the photoelectrocatalytic (PEC) platform based on nano‐Ni–P‐modified TiO_2_ nanorod photoanodes and nano‐Ni–P cathodes has achieved efficient and selective generation of H_2_ and formic acid from PET plastics. The use of heterojunctions as electrode pole materials has facilitated the upcycling of waste plastics. For instance, the visible light‐activated Fe_2_O_3_/Ni(OH)_*x*_ photoanode has been used for the PEC‐catalyzed conversion of PET plastic to value‐added chemicals such as formic acid/formate and H_2_ fuel without CO_2_ generation (**Figure** **11**a).^[^ ^179^ ^]^ Obviously, FA was determined as the main oxidative product (Figure 11b,c), and the FE of FA declined from ≈100% to below 80% with the increased potential (Figure 11d). In contrast, the decoration of Ni(OH)_*x*_ effectively solved the intrinsic sluggish reaction kinetics of the bare Fe_2_O_3_ photoanode (Figure 11e). The involvement of light energy and semiconductor catalysts can reduce the electrical energy consumption in the electrocatalytic process and improve the selectivity for the target products, while the separation and transport efficiency of the carriers in the PEC process could be improved by adjusting the bias voltage.

**Figure 12:**
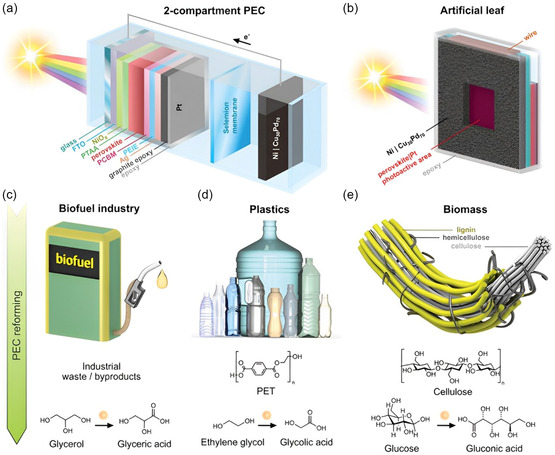
a) Schematic depictions of the Cu_30_Pd_70_|perovskite|Pt PEC waste‐reforming system in two compartment, and b) standalone “artificial leaf” configurations. c) Byproducts of the biofuel industry such as glycerol. d) Plastics such as PET and its monomer EG. e) Components of lignocellulosic biomass such as cellulose and its monomer glucose.^[^ ^180^ ^]^ Copyright 2022, Wiley‐VCH.

The design and preparation of devices is a critical step in the evolution from laboratory to industrialization, in order to explore the potential of PEC devices for waste conversion. A recent study has successfully developed a Cu_30_Pd_70_|perovskite|Pt composite PEC system, achieved by combining bimetallic Cu_30_Pd_70_ alloy microﬂowers (MFs) as photoanode with a lead‐halide perovskite photocathode and using Pt as hydrogen evolution catalyst (**Figure** **12**).^[^ ^180^ ^]^ A dual‐chamber and “artificial leaf” structure was used for the conversion of glycerol (Figure 12c), PET plastics (Figure 12d), and biomass‐derived substrates (Figure 12e) to clean H_2_ and industrially relevant chemicals. This PEC platform is 10^2^–10^4^ times more efficient than conventional photocatalytic conversion, providing a proof‐of‐concept alternative to the photoreforming platform, which more closely matches the performance and versatility required for commercially viable waste utilization.

Additionally, by implementing cascade reaction processes, innovative upcycling of waste plastics could be achieved, rather than solely relying on the direct degradation of raw materials into high‐value chemicals. The recycling of PE plastics has proven to be a significant challenge due to their high chemical stability. However, a preoxidative depolymerization strategy has been proposed, converting PE plastics into organic acids through the use of dilute nitric acid as oxidizing agent. These organic acids can then couple with photocatalytic or electrocatalytic decarboxylation reactions to produce hydrocarbons, with H_2_ and CO_2_ byproducts, ultimately forming valuable products.^[^ ^62^ ^]^ Using TiO_2_ or carbon nitride photocatalysis, the selectivity of the products can be finely tuned to produce alkanes such as ethane and propane, while electrocatalysis at carbon electrodes produces olefins like ethylene and propylene. Notably, this tandem upcycling pathway for plastics has the potential to produce valuable chemicals and high‐calorific‐value hydrocarbons using sunlight or renewable electricity. Moreover, the selectivity of products could be addressed to some extent by fine tuning the voltage of the electrolytic cell and the crystal orientation of the catalyst.

So far, although most chemical products through the upcycling process of waste plastics may still be relatively expensive compared to fossil fuels, plastics can be considered as valuable feedstock resources toward the end of their life, especially considering fossil fuels as a nonrenewable resource. Therefore, electrocatalytic upcycling of postconsumer plastics shows great promise.

## 4 Conclusion and Outlook

The massive accumulation of waste plastics has triggered a global environmental and ecological crisis, highlighting the need for new strategic approaches to resourceful upcycling of used plastics. The rapid advancements in photocatalytic and electrocatalytic technologies across multidisciplinary fields and the active exploration in the field of postconsumer plastics upcycling in recent years demonstrate that photo‐ and electrocatalysis will play a key role in addressing the plastics problem, reducing GHG to combat climate change and acquiring high‐value‐added products and clean energy. In this review, we present a summary and discussion of recent advances in waste plastic upcycling and energy generation through photo‐ and electrocatalysis degradation platforms, as well as innovative design of materials and technology processes. Conventional waste plastic conversion strategies often require extreme reaction conditions such as high temperature and pressure to synthesize high‐value‐added chemical products, leading to large amounts of CO_2_ release that contradict the principles of carbon cycle economy and sustainable circular development strategies. In contrast, photocatalytic and electrocatalytic waste plastic upcycling have the opportunity to operate under mild conditions, which are effective alternative pathways to traditional plastic recycling technologies. Although these technologies are still in their infancy and there is still much room for improvement in terms of product selectivity, singularity, and yield, encouragingly, recent research has shown that both technologies have great potential in upcycling plastics.

Although some progress has been made in the photocatalytic upcycling of plastics, further efforts are necessary in more directions to achieve successful technological transformation of various waste plastics. The following recommendations are now given.

As the central player in photo‐ and electrocatalytic process, catalysts are inherently complex. Consequently, the optimal regulation of catalysts to overcome scientific challenges is a key concern for chemists during the catalysts design and preparation processing. In particular, for light‐driven waste plastics upcycling, photocatalysts with suitable energy band positions and efficient activation polymer capability must be designed. This involves construction of heterojunctions, doping heteroatoms, and regulation the exposure of nanomaterial crystalline surface. Alternatively, for electrocatalysis cases of plastics upcycling, selecting electrocatalysts guided by the intrinsic properties of various metals with different activation capabilities for chemical bonds is crucial to identify suitable catalysts as required.

More importantly, the precise recognition of key active sites in the designed catalysts is extremely important and a great challenge, which will pave the way for the ensuing study of the reaction mechanism. A variety of research methods are candidates for the characterization and identification of active sites: spectroscopic methods are used to determine the types of bonds and chemical states on the catalyst surface to obtain the nature of the active sites; surface analysis techniques are used to provide the morphology, thickness, and crystal structure of the catalyst surface to locate the active sites; and theoretical calculation methods such as density functional theoretical can be used to determine the active sites by calculating the interactions between atoms on the catalyst surface. These research methods are combined with each other to obtain more comprehensive information on the characterization of active sites.

Microkinetic models and quantitative structure–activity relationships have far‐reaching implications for the design and preparation of catalysts as well. The intramolecular interactions and rigid framework structure of plastic polymers often result in poor contact between the polymer and the active sites of catalysts. Therefore, special attention should be given to improving the accessibility of plastic polymer substrates to the catalytic active sites. Since the catalyst plays a pivotal role in the initial stage of the catalytic reaction, mutual contact is fundamental for intermolecular interactions requires. Resolving the issue of direct contact between catalysts and substrates is crucial in improving the catalytic efficiency, ultimately influencing the resulting product selectivity in plastics upcycling. Finally, a judicious catalyst design can significantly enhance product yield, selectivity, and stability.

Whether photocatalytic or electrocatalytic waste plastic resourceful upgrading and recycling, we should not only focus on the innovative development of its process technology, but also focus on the study of the reaction mechanism involved in the catalyst at the molecular scale, including insight into the structure and performance relationship and the catalytic reaction pathways. This will be a very important step in the study of plastic resources upcycling process after the identification and confirmation of catalytically active sites. Despite the progress already made in this area, there is still a great deal of work to be done. One possible solution could be to utilize machine‐assisted techniques in order to better elucidate research protocols and monitor changes in energy. In order to better assess the degradation and recycling capabilities of the system, it will also be necessary to incorporate real plastics into the research system in addition to constructing model scenarios. With some of the recently available breakthroughs, the lack of standardized rules in the testing protocols of the products has hindered accurate performance comparisons between the evaluation results obtained from different research efforts.

The widely used polyolefin polymer plastics, such as PE and PP, which account for half of all plastics, have been less frequently reported on in regard to photocatalytic and electrocatalytic upcycling. This is likely due to their high chemical inertness, making their degradation extremely difficult. Thus far, the most effective treatment method for catalyzing these polymers (LDPE, high‐density polyethylene (HDPE), and PP) is pyrolysis, which produces liquid hydrocarbons (HCs). To improve the upcycling of these plastics, a preliminary chemical depolymerization process through pretreatment could be considered. Additionally, as plastics mainly consist of a polymeric structure, future research could focus on the conversion strategies for natural polymer molecules, such as biomass.^[^ ^181^ ^]^

Resourceful upcycling of recycled plastics on a global scale is undoubtedly a challenging topic. However, we firmly believe that with the constant research and development of innovative process technologies, as well as novel catalysts, the photocatalytic upcycling of waste plastics will play a vital role in transitioning to a zero‐carbon economy and in the cost‐effective production of high‐value‐added chemical products.

## Conflict of Interest

The authors declare no conflict of interest.
